# The Role of Ferroptosis in Diabetes Pathogenesis: Therapeutic Implications of Hydrogen Sulfide and Its Reactive Metabolites

**DOI:** 10.3390/antiox15030369

**Published:** 2026-03-13

**Authors:** Vesna Otasevic, Milica Markelic, Marko Miler, Nevena Savic, Ksenija Velickovic, Andjelija Gudelj, Ilijana Grigorov, Ana Stancic

**Affiliations:** 1 Department of Molecular Biology, Institute for Biological Research “Siniša Stanković”, National Institute of Republic of Serbia, University of Belgrade, 11000 Belgrade, Serbia; 2 Department of Cell and Tissue Biology, Faculty of Biology, University of Belgrade, 11000 Belgrade, Serbia; 3 Department of Citology, Institute for Biological Research “Siniša Stanković”, National Institute of Republic of Serbia, University of Belgrade, 11000 Belgrade, Serbia

**Keywords:** ferroptosis, diabetes, hydrogen sulfide, iron, lipid peroxidation, persulfidation

## Abstract

Ferroptosis, a regulated form of cell death characterized by iron-dependent lipid peroxidation, emerged as an important contributor to the pathogenesis of diabetes and its complications. Impaired glucose and iron metabolism, and increased oxidative stress, predispose cells—particularly pancreatic β-cells and vascular tissues—to ferroptotic cell death, contributing to β-cell dysfunction, insulin resistance, and the progression of diabetic complications. Hydrogen sulfide (H_2_S), an important gasotransmitter, plays a pivotal role in regulating various pathophysiological processes by interfering with key cellular signaling pathways, including those related to cell death. In the context of ferroptosis, H_2_S exerts protective effects by activating the nuclear factor erythroid 2-related factor 2/glutathione peroxidase 4/glutathione (Nrf2/GPX4/GSH) axis, enhancing cellular antioxidative defenses and inhibiting lipid peroxidation. Furthermore, H_2_S modulates key regulators of iron homeostasis and lipid metabolism, including hepcidin, ferritin, and the cystine/glutamate antiporter system (xCT) antiporter, further attenuating ferroptosis. Exogenous administration of H_2_S can reverse ferroptosis-induced cellular injury in several pathological settings and improve metabolic outcomes in diabetic models. These findings suggest that targeting H_2_S signaling is a promising therapeutic strategy to inhibit ferroptosis and mitigate diabetes-related organ dysfunction. This review summarizes current insights into the molecular interplay between H_2_S and diabetes-related signaling pathways, primarily ferroptosis, emphasizing the antiferroptotic therapeutic potential of H_2_S-based interventions for the prevention and treatment of diabetic complications.

## 1. Introduction

Diabetes mellitus is a chronic metabolic disease characterized by high blood glucose levels resulting from insufficient insulin production, impaired insulin action or both. It is associated with disturbances in the metabolism of the main energy substrates, glucose and lipids, leading to hyperglycemia and hyperlipidemia, which are linked to increased oxidative stress and inflammation—both critical factors in the development of diabetic complications [1,2,3]. Diabetes is primarily classified into type 1 diabetes (T1D) and type 2 diabetes (T2D), distinguished by their underlying mechanisms of impaired glucose regulation. Although both forms ultimately involve inadequate insulin action, their origins and progression differ. Diabetes affects people of all ages, but certain groups are at higher risk; T1D is more commonly diagnosed in children and young adults and accounts for about 10% of all diabetes cases, while T2D is more common in middle-aged and older individuals, particularly those with a sedentary lifestyle [4]. The disease is more prevalent in low- and middle-income countries with limited healthcare and preventive measures, and in urban areas compared to rural areas due to dietary habits and lifestyle.

The global prevalence of diabetes is rising at an epidemic rate [5,6], affecting 11.1% of adults (589 million) worldwide in 2024, with nearly half undiagnosed. The number is projected to exceed 850 million by 2050 [7]. This increase presents a major socioeconomic and healthcare challenge due to the high cost of treatment (11.9% of the global healthcare budget), with annual expenditures exceeding $1 trillion [7]. Diabetes is one of the leading causes of mortality worldwide, and diabetes-induced cardiovascular disease is the primary cause of death among people with diabetes [8]. The severity and duration of uncontrolled diabetes lead to life-threatening complications in various tissues, such as neuropathy, nephropathy, retinopathy, and cardiovascular diseases. The danger of diabetes lies in its silent progression; many complications develop over years before symptoms appear, making early detection and treatment crucial. Despite available treatment options, diabetes remains a major health threat, and there is a growing need for new therapies. The primary limitation of current therapies is that they address the symptoms and complications rather than the root cause of the disease. Therefore, ongoing research aims to develop more effective approaches, including early detection and innovative treatment strategies, to reduce the impact on individuals and society. It is essential to reveal and understand the complex pathogenesis of the disease.

Cell death plays an important role in the development and progression of both T1D and T2D, as it contributes to β-cell failure and diabetes-related organ injuries. The loss of β-cells leading to T1D occurs silently, with hyperglycemia typically appearing only after most β-cells (~80%) have been destroyed, which limits therapeutic options [9,10,11]. Early detection of β-cell death would allow interventions before hyperglycemia develops, when β-cell mass is still preserved. Notably, immunotherapy is most effective in patients who retain some β-cell function [11,12,13]. To date, several types of β-cell death have been described in diabetic conditions, including necrosis, apoptosis, and autophagy [14,15,16,17,18]. Recent studies, including ours, have begun to reveal a link between diabetes-induced cell death and ferroptosis in both the pancreas [19,20] and tissues susceptible to diabetic complications [21,22,23,24,25,26,27]. Ferroptosis is a relatively newly defined, iron-dependent form of regulated cell death characterized by the accumulation of lipid peroxides to lethal levels in cell membranes, resulting in oxidative cell damage [28]. It is triggered by dysfunction of the cystine/glutamate antiporter system (xCT, solute carrier family 7 member 11, SLC7A11), glutathione (GSH) depletion, iron overload, and inactivation of glutathione peroxidase 4 (GPX4), a membrane-associated isoform of the GPX family specialized in removing lipid peroxides [29]. Ferroptosis is genetically, biochemically, and morphologically distinct from other types of cell death [30,31], which allows for specific detection and modulation of this form of cell death. In recent years, ferroptosis has been found to be involved in diabetes and its multiple complications [21,22,23,24,25,26,27], and its pharmacological modulation has emerged as a promising therapeutic strategy for diabetes and related pathologies.

In this context, alongside the complex interplay of biochemical pathways involved in diabetes progression and its associated complications, hydrogen sulfide (H_2_S), a gasotransmitter endogenously produced in the body, has emerged as a molecule of substantial interest. The discovery that H_2_S is synthesized not only in pancreatic β-cells but also in key insulin-target organs such as the liver, adipose tissue, and skeletal muscle highlights the potential role of H_2_S in regulating insulin signaling and glucose metabolism [32,33,34]. Its influence extends to the pathophysiological context of diabetes, where altered H_2_S levels have been observed in both diabetic animals [35,36,37] and patients [36,38]. Numerous studies consistently link reduced H_2_S levels in plasma and tissues to the metabolic complications associated with diabetes. Consequently, the use of H_2_S donors has emerged as a promising therapeutic strategy to address diabetes-related conditions such as endothelial dysfunction [37,39,40], atherosclerosis, retinopathy [41,42,43,44], nephropathy [45,46], cardiomyopathy [47,48], and cognitive deterioration [49]. However, the role of H_2_S in regulating β-cell function and survival remains controversial and context-dependent. Factors such as age, diabetes type/stage, experimental models, the type of H_2_S donor used, and dosage/duration of treatment contribute to these discrepancies. An intricate process through which H_2_S exerts its biological effect is persulfidation, a posttranslational modification in which a persulfide group is added to specific cysteine residues on target proteins [50,51,52]. This modification can profoundly affect protein function, signaling pathways, and cellular defense mechanisms. In the diabetic milieu, perturbations in persulfidation dynamics may be pivotal in the onset and development of various diabetic complications.

Regarding the interference between H_2_S and ferroptosis signaling pathways, data show that exogenous administration of H_2_S and its donors reverses ferroptosis-induced cell injury in various pathological contexts, ameliorates cardiac and renal injury, and improves metabolic outcomes in diabetic models [24,42,47,53,54]. Notably, recent studies indicate that H_2_S can modulate ferroptosis by influencing GSH metabolism, iron handling, and lipid peroxidation [55,56,57]. These findings suggest that targeting H_2_S signaling represents a promising therapeutic strategy to inhibit ferroptosis and alleviate diabetes-related organ dysfunction. This review summarizes current knowledge on the interplay between H_2_S signaling and ferroptotic pathways in diabetes. We discuss current findings on the involvement of ferroptosis and disturbances of H_2_S signaling in diabetic etiology and pathogenesis; the interference between H_2_S and ferroptosis signaling pathways based on the regulatory role of H_2_S over antioxidative defense, iron metabolism, and inflammation (all closely related to ferroptosis); and emphasize the therapeutic potential of H_2_S-based interventions for the prevention and treatment of diabetes and its related disorders. By consolidating current knowledge and identifying future research directions, we aim to highlight the significance of these sulfur-based mechanisms in addressing a global health challenge.

## 2. Diabetes: Classification and Pathological Characteristics

Diabetes is a complex metabolic disorder that develops as a result of impaired insulin secretion and/or action. T1D results from autoimmune destruction of pancreatic β-cells, causing severe insulin deficiency, while T2D is characterized by insulin resistance and progressive β-cell dysfunction, closely linked to lifestyle factors such as obesity and physical inactivity. Insulin resistance is defined as the inability of peripheral target tissues—primarily the liver, adipose tissue, and skeletal muscle—to respond to insulin. In the early stages of insulin resistance, β-cells compensate by increasing insulin secretion to maintain blood glucose stability. As the disease progresses, β-cells can no longer secrete enough insulin to compensate for abnormally high blood glucose, either due to β-cell dysfunction and/or a decrease in β-cell mass, leading to the progression from insulin resistance to prediabetes and ultimately to T2D [58,59].

Although the underlying causes differ, the key pathophysiological feature linking both T1D and T2D is β-cell dysfunction and/or β-cell loss [60]. Impaired glucose and lipid metabolism in both types of diabetes leads to hyperglycemia and hyperlipidemia, accompanied by inflammation. Additionally, obesity-related hyperlipidemia is a causative factor for insulin resistance that precedes β-cell failure. Excess lipids are deposited in non-adipose tissues, mainly skeletal muscle and liver, disrupting insulin signaling pathways. Decreased glucose uptake and increased hepatic glucose production further raise circulating glucose and basal insulin levels. Elevated insulin levels promote lipid deposition, aggravating insulin resistance and creating a vicious cycle; elevated glucose and lipids contribute to β-cell injury, producing a state often referred to as hyperglucolipotoxicity, which decreases both the secretory function and number of β-cells, further increasing blood glucose [61]. Chronic hyperglycemia and hyperlipidemia lead to metabolic complications in other tissues and the development of various diabetes-related pathologies, commonly classified as microvascular and macrovascular complications. Microvascular complications include retinopathy, nephropathy, and neuropathy, while macrovascular complications involve cerebrovascular, cardiovascular, and peripheral vascular diseases.

### Oxidative Stress in the Diabetic State: Causes and Consequences

Oxidative stress is a condition marked by increased accumulation of reactive oxygen species (ROS). The main intracellular source of ROS is the mitochondrial electron transport chain (ETC), where a small fraction of electrons (approximately 0.2–2%) escape normal electron transfer and react directly with oxygen, generating superoxide anion or its derivative, hydrogen peroxide (H_2_O_2_) [62]. In addition to mitochondrial respiration, several enzymes and metabolic reactions naturally produce superoxide and H_2_O_2_ under physiological conditions. At physiological concentrations, ROS serve as important regulators of many intracellular signaling pathways. Physiological ROS levels are maintained by antioxidative defense mechanisms. The biological antioxidative system includes enzymatic and non-enzymatic components that act together to neutralize ROS. Some of the most important endogenous antioxidative enzymes are catalase (CAT), superoxide dismutase (SOD), and glutathione peroxidase (GPX), while the most important non-enzymatic antioxidant is GSH, a cofactor for GPX [63]. Additionally, the thioredoxin (TRX)/thioredoxin reductase (TR), peroxiredoxin, and glutaredoxin systems are involved in redox regulation in the pancreas [64].

Excess accumulation of ROS results from either increased production or decreased removal and characterizes many pathological states, including diabetes. The main pathway of glucose metabolism under physiological conditions is glycolysis, which supplies human cells with energy as ATP through ETC activity and oxidative phosphorylation (OXPHOS). In diabetes, increased glucose flux enhances ROS production via the ETC. Elevated glucose levels also activate several alternative metabolic pathways—including protein kinase C (PKC), polyol, and hexosamine pathways, which are closely linked to glycolysis—and spontaneous glucose reactions, protein glycation, and advanced glycation end product (AGE) formation, all of which contribute to oxidative stress.

Activation of the PKC pathway increases diacylglycerol levels and activates several pro-oxidant enzymes, such as nitric oxide synthase (NOS), xanthine oxidase (XO), and lipoxygenases (LOX), thereby enhancing ROS generation [65]. The polyol pathway produces sorbitol and fructose, disturbing cellular redox balance by altering NADPH and NADH. The hexosamine pathway increases protein glycosylation, endoplasmic reticulum (ER) stress and insulin resistance [66]. Non-enzymatic glycation of proteins produces Amadori intermediates and AGEs, whose interaction with the receptor for advanced glycation end products (RAGEs) further amplifies oxidative stress and inflammatory signaling [67,68].

Chronic hyperlipidemia, another hallmark of diabetes, also induces persistent ROS production. The mechanisms involve elevated mitochondrial respiration due to increased fatty acid β-oxidation and flux through the ETC, and increased activity of NADPH oxidase (NOX) in a PKC-dependent manner [69].

The third pathological hallmark of diabetes, inflammation, is also closely linked to oxidative stress. ROS activate proinflammatory transcription factors, such as nuclear factor kappa B (NF-κB), promoting cytokine release and recruitment of immune cells, which further increase ROS generation. This establishes a self-reinforcing cycle resulting in chronic systemic inflammation in the diabetic state.

Beyond increased ROS production, oxidative stress in diabetes also results from impaired removal due to compromised antioxidative defense. Reduced plasma or serum total antioxidative status and decreased levels of specific antioxidants such as ascorbic acid, SOD, GPX, and catalase activity, and free radical scavenging activity, have been reported in diabetic subjects [70,71]. Moreover, the degree of antioxidant impairment correlates with the severity of hyperglycemia, indicating that elevated glucose is a driving force behind antioxidant system failure [72].

**Figure 1:**
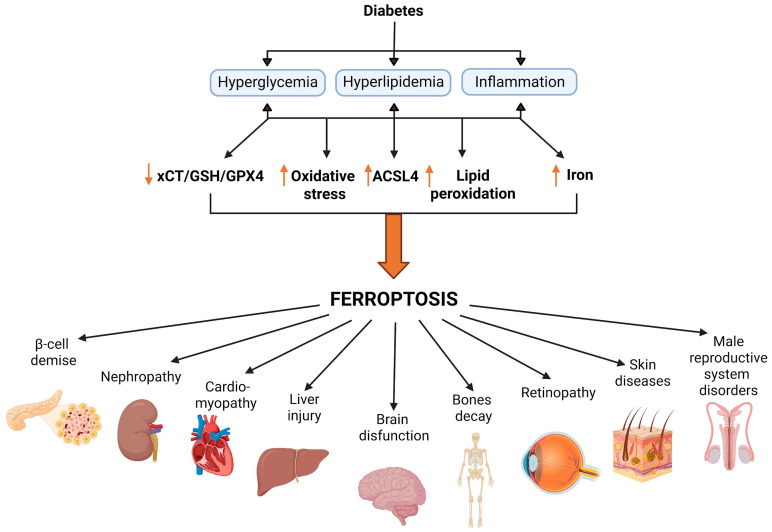
Pathophysiological mechanisms of ferroptosis in diabetes and its complications. Diabetes-associated metabolic disturbances, including hyperglycemia, hyperlipidemia, and chronic inflammation, lead to excessive production of reactive oxygen species, inducing oxidative stress. In addition, diabetic metabolic disturbances increase the labile iron pool and ACSL4 levels, and impair the xCT/GSH/GPX4 axis, resulting in increased lipid peroxidation and ultimately ferroptosis. Ferroptosis contributes to pancreatic β-cell death and plays a pivotal role in the development and progression of diabetic complications, including nephropathy, cardiomyopathy, retinopathy, and dysfunction of the liver, brain, bones, skin, and male reproductive system. Created in BioRender. Vidakovic, M. (2026) https://BioRender.com/2oq0pif (accessed on 29 December 2025).

The consequences of oxidative stress in diabetes are extensive, affecting multiple redox-sensitive intracellular signaling pathways, including c-Jun N-terminal kinase/stress-activated protein kinase (JNK/SAPK), NF-κB, and mitogen-activated protein kinase (MAPK) pathways (such as p38 MAPK and extracellular signal-regulated kinase, ERK), and PKC pathways [73]. This leads to dysfunction and ultimately the death of β-cells and cells in insulin-responsive tissues. Several types of cell death have been identified as contributing to the etiology and pathogenesis of diabetes. Oxidative stress acts as a common mechanism across different types of regulated cell death implicated in diabetes. Apoptosis is considered the predominant form of β-cell death in both types of diabetes [74], while apoptosis, necroptosis, and an imbalance between autophagy and apoptosis play important roles in the progression of diabetic complications in the heart, retina, kidneys, and nervous system [75,76]. More recently, evidence from our group and others indicates that ferroptosis, driven by iron-dependent lipid peroxidation of polyunsaturated fatty acids in membranes and xCT/GSH/GPX4 failure, contributes to pancreatic and peripheral tissue damage in diabetes (Figure 1) [19,20,21,22,27].

## 3. Ferroptosis: Key Signaling Pathways and Molecular Players

**Figure 2:**
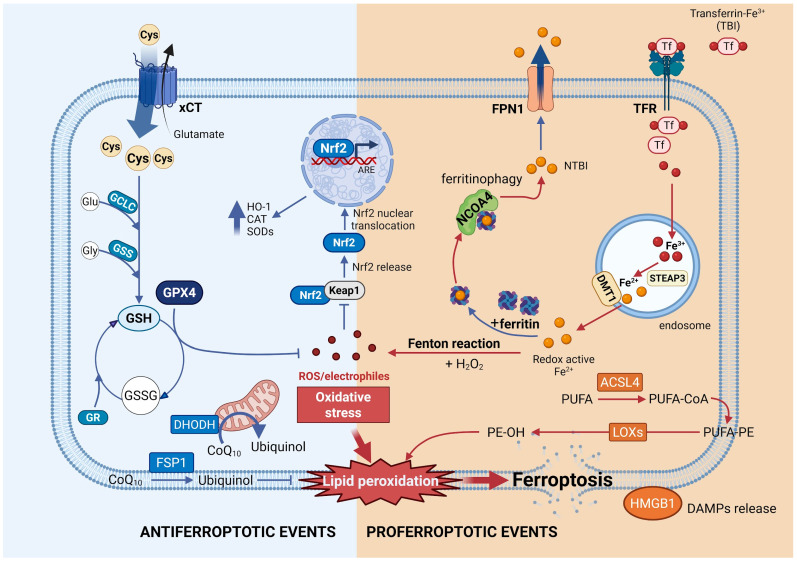
The main pro- and antiferroptotic cellular pathways. The central event in the lipid peroxide production pathway is the Fenton reaction, which occurs between Fe^2+^ and H_2_O_2_. Iron is imported into the cell as an iron-loaded transferrin-transferrin receptor 1 (TFR1) complex via receptor-mediated endocytosis. In the endosome, an acidic environment, free Fe^3+^ is converted to Fe^2+^ by the transmembrane metalloreductase, six-transmembrane epithelial antigen of the prostate 3 (STEAP3), and released into the cytoplasm via the divalent metal transporter 1 (DMT1). Fe^2+^ is stored in the cytoplasm by ferritin. Degradation of ferritin through ferritinophagy, mediated by the cargo receptor nuclear receptor coactivator 4 (NCOA4), leads to the release of iron, which can be exported from the cell by ferroportin 1 (FPN1). Otherwise, there is increased sensitivity to ferroptosis. The enzymatic pathway of lipid peroxidation involves enzymes that metabolize arachidonic acid and polyunsaturated fatty acids (PUFAs), with acyl-CoA synthetase long chain family member 4 (ACSL4) and lipoxygenases (LOXs) playing key roles. The main regulatory pathway of ferroptosis involves the cysteine-GSH-GPX4-lipid peroxide axis. Cysteine is transported into the cell via the cystine/glutamate antiporter system (xCT) in its oxidized form (cystine). One of the main roles of cysteine is the synthesis of glutathione (GSH), in which the rate-limiting step is catalyzed by glutamate-cysteine ligase (GCL), followed by the step involving glutathione synthetase (GSS). GSH serves as a cofactor for glutathione peroxidase 4 (GPX4), which reduces lipid peroxides to their alcohol form, thereby oxidizing GSH to GSSG. GSSG is reduced back by the enzyme glutathione reductase (GR). Alternatively, lipid peroxides can be reduced by ubiquinol in the membrane compartments of the cell. Ubiquinone produced in this process is reduced back to its alcohol form by the action of ferroptosis-suppressor protein 1 (FSP1) and dihydroorotate dehydrogenase (DHODH). Most ferroptosis-related molecules, both antioxidative and those involved in iron metabolism, are regulated by nuclear factor erythroid 2-related factor 2 (Nrf2), a central redox-sensitive transcription factor. Red arrows represent proferroptotic pathways, while blue arrows represent antiferroptotic pathways. Created in BioRender. Vidakovic, M. (2026) https://BioRender.com/66ebhfh (accessed on 29 December 2025).

Ferroptosis is a distinct form of regulated cell death characterized by its dependence on iron and the accumulation of lipid peroxides (Figure 2). It differs from other cell death modalities in its biochemical mechanisms, key triggers and effectors, and morphological features. Since its identification as a novel cell death modality by the Stockwell group in 2012, evidence supporting ferroptosis as a unique process has grown considerably [28]. The central event in ferroptosis is the excessive and uncontrolled accumulation of lipid peroxides, which results from an imbalance between their formation and removal. Two primary mechanisms drive lipid peroxide formation: enzymatic and non-enzymatic pathways [77]. The enzymatic pathway involves enzymes that metabolize arachidonic acid and polyunsaturated fatty acids (PUFAs), with LOXs playing a key role. The non-enzymatic process involves redox chemistry, initiated when ROS, reactive nitrogen species (RNS), or reactive lipid species (RLS) abstract a hydrogen atom from a PUFA, generating a lipid radical (L^∙^) [77]. Among these, the hydroxyl radical (^∙^OH) and the hydroperoxyl radical (^∙^OOH)—produced through the Fenton reaction between ferrous iron (Fe^2+^) and H_2_O_2_—are particularly potent initiators [77]. Regardless of the initial pathway, lipid peroxidation proceeds through a propagation phase and a termination phase [78]. During propagation, L^∙^ reacts rapidly with oxygen to form a lipid peroxy radical (LOO^∙^), which then abstracts a hydrogen atom from another lipid molecule, resulting in a new L^∙^ and a lipid hydroperoxide (LOOH) [77]. This chain reaction continues until end products are formed. The lipid peroxides generated in this phase can further convert into hydroxy fatty acids or reactive aldehydes, which themselves can perpetuate lipid peroxidation [79].

The exact mechanism by which lipid peroxidation culminates in ferroptotic cell death remains incompletely understood. It is hypothesized that this process involves both membrane damage and activation of downstream signaling cascades [80,81,82]. Peroxidation of membrane lipids impairs membrane integrity, alters physical properties, reduces fluidity, increases permeability, disrupts ion gradients, and slows lateral diffusion [83,84,85]. Additionally, secondary products of PUFA peroxidation can affect protein structure and function, serving not only as primary biomarkers for lipid peroxidation assessment but also as contributors to multiple signaling pathways related to cell death [86,87]. In particular, malondialdehyde (MDA) and 4-hydroxynonenal (4-HNE) are the most prevalent and extensively studied products associated with various types of cell death [88,89]. These aldehydes have also been found to accumulate in several ferroptosis models [90,91]. MDA and especially 4-HNE act as toxic second messengers of oxidative stress, propagating damage throughout the cell by forming harmful protein adducts and disrupting critical homeostatic and antioxidant systems, ultimately executing the ferroptotic cell death program. Moreover, lipid peroxidation in ferroptosis is facilitated by acyl-CoA synthetase long chain family member 4 (ACSL4), an acyl CoA synthetase enzyme that acylates PUFAs and generates fatty acyl-CoA esters, which are then transesterified into phospholipids [92].

Under physiological conditions, the lipid peroxidation cascade is mitigated by GPX4, a unique antioxidative enzyme that reduces LOOH to the corresponding alcohols using GSH as a cofactor. The cysteine-GSH-GPX4-lipid peroxide axis represents the central regulatory framework of ferroptosis signaling [93]. GPX4 is considered the primary enzymatic defense against ROS-mediated membrane peroxidation, and thus ferroptosis, due to its strong membrane association and close proximity to phospholipid peroxide substrates [93,94,95]. Supporting this, increasing evidence shows that direct inhibition of GPX4 by RAS-selective lethal 3 (RSL3) treatment or GPX4 knockout in mice results in severe membrane lipid peroxidation and ultimately ferroptotic cell death [96,97,98]. GSH, a tripeptide, is essential for redox homeostasis, mainly due to the reactive thiol group of its cysteine residue. In the context of ferroptosis, GSH is crucial not only for GPX activity but also for modulation of LOX activity and subcellular localization [99], and involvement in the redox cycling of Fe^2+^ [82,100]. Depletion of GSH increases LOX activity and membrane association, and increases the availability of substrates for the Fenton reaction. In addition, GSH acts as a cofactor for glutathione S-transferase (GST) in the detoxification of ROS and xenobiotics [101].

In addition to GPX4, ferroptosis suppressor protein 1 (FSP1) plays a critical role in mitigating ferroptosis by limiting lipid peroxide accumulation. Initially described as apoptosis-inducing factor mitochondria-associated 2 (AIFM2), this protein was reclassified as FSP1 after its role in ferroptosis resistance was discovered [102,103]. When GPX4 is inactivated or absent, FSP1 protects cells by catalyzing the NAD(P)H-dependent reduction of ubiquinone (coenzyme Q10) to its reduced, antioxidative form ubiquinol (CoQ10-H_2_), which neutralizes LOO^∙^ [103,104]. FSP1 also promotes ferroptosis defense by recruiting the endosomal sorting complex required for transport-III (ESCRT-III) complex, which repairs oxidatively damaged cell membranes [105], and by regenerating lipid-soluble antioxidants such as vitamins E and K [103,104,105,106,107]. Recent research on FSP1 inhibition has identified a novel class of compounds, 3-phenylquinazolinones, that promote the phase separation of FSP1 into inactive molecular condensates. This disruption sensitizes cells to ferroptosis-inducing agents, highlighting FSP1 as a promising therapeutic target in cancer treatment [108].

Another key aspect of ferroptosis that represents a promising target for its modulation is iron metabolism. Iron accumulation is a hallmark of ferroptosis due to its central role in triggering lipid peroxidation via the Fenton reaction. Therefore, disturbances in cellular iron homeostasis that favor iron accumulation are major drivers of ferroptosis [109,110,111,112]. Tight regulation of intracellular iron homeostasis is crucial for inhibiting ferroptosis and maintaining normal cellular functions [113]. Iron homeostasis is a dynamic equilibrium among iron uptake, storage, utilization, and export. Cellular iron uptake occurs primarily via transferrin receptor 1 (TFR1), located on the cell membrane [109]. In this pathway, transferrin-bound iron (TBI) binds to TFR1, forming a transferrin–iron complex that is internalized by endocytosis [114,115]. During transport, transferrin keeps the iron in a non-reactive, exchange-inhibited state [116]. Once internalized, Fe^3+^ is converted to Fe^2+^ by the metalloreductase (STEAP3), contributing to the labile iron pool—a redox-active iron fraction whose expansion accelerates lipid peroxidation [109,116,117,118]. Although the TFR1 pathway predominates, alternative iron uptake mechanisms exist. Under conditions of iron overload, transferrin can become saturated, resulting in non-transferrin-bound iron (NTBI) in circulation [109]. Certain cell-surface ferrireductases reduce NTBI to Fe^2+^, which can then be imported into cells by NTBI-specific transporters such as divalent metal transporter 1 (DMT1) or members of the ZIP family [116,117]. Intracellular iron is primarily stored in ferritin, a complex of ferritin heavy chain (FTH1), ferritin light chain (FTL) and Fe^3+^, thereby preventing iron-mediated oxidative damage [119,120]. Ferritin is selectively degraded through ferritinophagy, a specialized autophagic process mediated by the cargo receptor nuclear receptor coactivator 4 (NCOA4), which releases stored iron for cellular use. Ferritinophagy is regulated by iron levels: it is promoted under iron-deficient conditions to release iron, whereas iron sufficiency promotes NCOA4 ubiquitination and proteasomal degradation, thus limiting ferritinophagy and favoring iron storage [121,122]. However, pathological upregulation of NCOA4 increases ferritin degradation and promotes ferroptosis [122]. Excess intracellular Fe^2+^ is oxidized to Fe^3+^ and exported from the cell via ferroportin (FPN), contributing to the maintenance of cellular iron balance [121,123]. Disruptions in this regulatory network collectively lead to elevated labile iron, increased lipid peroxidation, and ferroptosis [124].

Intrinsic cellular defenses against ferroptosis include the antioxidative transcription factor nuclear factor erythroid 2-related factor 2 (Nrf2), which is a key player in both the initiation and consequences of this type of cell death [125]. Nrf2 regulates redox balance and cellular protection against damage [126,127]. Under physiological conditions, Nrf2 is sequestered in the cytoplasm by its inhibitor, kelch-like ECH-associated protein 1 (Keap1). Upon oxidative stress, Nrf2 translocates to the nucleus, where it induces the expression of various detoxifying genes involved in antioxidant defense, GSH metabolism (xCT, GPX4, and glutathione reductase, GR), iron handling (FTH1, TRF, and heme oxidase, HO) [128,129,130,131], and lipid metabolism [132,133]. Activation of Nrf2 signaling serves as an intrinsic defense against ferroptosis and represents a therapeutic strategy in several pathological contexts, including diabetes. Conversely, suppressing Nrf2, whether through inactivation, pharmacological inhibition, or gene knockdown, sensitizes cells to ferroptosis [133].

Recently, dihydroorotate dehydrogenase (DHODH), a mitochondrial inner membrane enzyme involved in de novo pyrimidine biosynthesis, has been identified as a key mitochondrial suppressor of ferroptosis [134]. DHODH catalyzes the oxidation of dihydroorotate to orotate while reducing ubiquinone (CoQ) to ubiquinol (CoQH_2_), thereby sustaining a mitochondrial antioxidant pool that limits lipid peroxidation within mitochondrial membranes. Importantly, DHODH functions independently of the cytosolic GPX4 system and represents a compartmentalized ferroptosis defense pathway.

Beyond redox-related mechanisms, ferroptosis is closely associated with inflammation. A bidirectional relationship exists in which ferroptosis activates inflammatory signaling, and inflammatory pathways in turn exacerbate ferroptosis. Several inflammatory pathways, including the Janus kinase/signal transducer and activator of transcription (JAK/STAT) pathway, NF-κB, the inflammasome, the cyclic GMP-AMP synthase–stimulator of interferon genes (cGAS-STING) pathway, and MAPK signaling, have been linked to ferroptosis [135,136,137]. Recent studies also highlight a significant role for high mobility group box 1 protein (HMGB1) in ferroptosis-associated inflammation. Ferroptosis inducers promote the translocation of HMGB1 from the nucleus to the cytosol and ultimately to the extracellular space, where it triggers a proinflammatory cascade upon binding with RAGE. Additionally, HMGB1 inhibition or RAGE deficiency attenuates the ferroptosis-induced inflammatory response in macrophages, indicating that targeting HMGB1 release may limit iron-driven inflammatory responses during ferroptosis [138]. 

Estrogen receptor alpha (ESR1) has recently been proposed as an additional negative regulator of ferroptosis, acting by modulating ferroptotic pathways, primarily through transcriptional activation of SLC7A1 [139]. ESR1 also facilitates the ubiquitination and degradation of the transferrin receptor CD71, limiting iron uptake and ferroptosis susceptibility, as demonstrated in breast cancer cells exposed to ionizing radiation and extended to broader cellular contexts [140]. Beyond oncology, ESR1 promotes an anti-ferroptotic hydropersulfide system in renal tubular cells of female mice, enhancing resistance to ischemia–reperfusion injury [141], while its knockdown induces ferroptosis markers such as reduced GPX4/SLC7A11 and elevated ACSL4 in uterine leiomyoma cells via Wnt/β-catenin inhibition [142]. These mechanisms highlight ESR1’s protective role against ferroptosis in both cancerous and non-cancerous tissues [141,143].

## 4. Ferroptosis in Pathological Conditions: Focus on Diabetes

Since the discovery of ferroptosis, its beneficial role in regulating cell mass under physiological conditions and its implications in various pathological settings have become increasingly evident. Extensive research in recent years has shown that ferroptosis is associated with cancer, neurodegenerative, cardiovascular, and hematologic diseases, and ischemia/reperfusion (I/R) injury in multiple organs, including the heart, brain, and kidney [98,138,144,145,146,147]. As noted above, a growing body of evidence also links ferroptosis to the onset and progression of diabetes and related metabolic disorders. Thus, modulating ferroptosis—either through targeted induction (e.g., in cancer) or prevention (e.g., in neurodegenerative, cardiovascular diseases and diabetes)—represents a promising strategy that complements existing apoptosis-targeting treatments and may improve disease outcomes. To date, numerous ferroptosis inducers and inhibitors have been developed to modulate this form of cell death. Their chemical structures, mechanisms of action, and applications in preclinical and clinical studies are summarized in Supplementary Table S1.

### 4.1. Ferroptosis in β-Cell Mass Reduction in Diabetes

Early evidence linking ferroptosis to diabetes pathophysiology emerged from studies investigating oxidative stress and iron metabolism in pancreatic β-cells. These cells are uniquely susceptible to oxidative damage due to low intrinsic antioxidative defense, high metabolic activity [148,149,150,151], and limited DNA repair efficiency in response to oxidative injury [152]. Ristow et al. [153] first demonstrated that frataxin deficiency, which leads to mitochondrial iron overload, causes β-cell death and diabetes in mice. Subsequent studies showed that iron overload exacerbates hyperglycemia and β-cell dysfunction [154,155], establishing the basis for examining ferroptosis as a mechanistic link.

A landmark study by Bruni et al. [156] showed that the viability and function of isolated pancreatic islets are severely compromised in the presence of ferroptosis inducers. They further reported that human islets isolated for transplantation are susceptible to erastin- and RSL3-induced ferroptosis, and that ferrostatin-1 (Fer-1), a canonical ferroptosis inhibitor, prevents this loss of function. Our research group has contributed substantially to identifying ferroptotic mechanisms in β-cells. In our recent in vitro study, we demonstrated for the first time that mimicking diabetic conditions with high glucose (HG), streptozotocin (STZ) or H_2_O_2_ increases cell death related to the accumulation of lipid peroxides in RIN-5F insulinoma cells [19]. This effect corresponded with increased accumulation of ROS, iron, and lipofuscin, inactivation of Nrf2, decreased GPX4 expression, and reduced mitochondrial membrane potential. Most importantly, this effect was abolished by Fer-1 treatment, which rescued cells from death, confirming ferroptosis as a mode of β-cell death under diabetic conditions in vitro. We further extended these findings in vivo, showing that Fer-1 protects pancreatic islets from STZ-induced injury by reducing macrophage infiltration and lipid peroxide accumulation while preserving the population of insulin-positive β-cells [19]. In this and our subsequent in vivo study, pharmacological inhibition of ferroptosis restored the Nrf2/xCT/GPX4 axis, preserved insulin secretion and β-cell viability, and normalized the ratio and distribution of α- and β-cells in islets, highlighting the therapeutic potential of targeting this pathway [20].

Our findings align with those of other authors and collectively indicate that, mechanistically, ferroptosis in β-cells is driven by dysregulated redox balance and iron handling. High glucose and corresponding pro-oxidative (lipofuscin, MDA and 4-HNE) and pro-inflammatory (increased HMGB1, cyclooxygenase-2, COX2 expression) conditions cause the accumulation of labile iron, upregulation of TFR1, and downregulation of FTH1, which contribute to iron-mediated ROS generation and lipid peroxidation-induced ferroptotic β-cell failure [157,158].

Moreover, we provide further insight into the regulation of iron homeostasis and lipid metabolism under diabetic conditions, identifying a new factor involved in ferroptosis sensitivity in pancreatic tissue—peroxiredoxin-2 (PRDX2) [159]. Although the PRDX family has already been shown to protect various cells from ferroptosis [160], to our knowledge, the role of PRDX2 downregulation in the ferroptosis of islet cells is demonstrated here for the first time in diabetes.

### 4.2. Role of Ferroptosis in Tissues Affected by Diabetic Complications

Beyond the pancreas, ferroptosis has been increasingly implicated in the pathogenesis of diabetic complications in multiple organs, including the kidney, heart, liver, brain, bone, skin, retina, and testis. Evidence from numerous in vitro and in vivo experimental models and human studies provides comprehensive insight into the mechanisms by which ferroptosis contributes to diabetes-associated damage in various tissues. Although diabetic complications differ in cellular composition and pathological manifestations among organs, they share several core features of ferroptotic cell death. These include hyperglycemia-driven disruption of iron homeostasis, leading to increased ROS production, mitochondrial dysfunction, impaired antioxidative defense, and enhanced lipid peroxidation. At the same time, ferroptosis exhibits pronounced tissue-specific characteristics determined by differential expression of iron metabolism regulators, antioxidative enzymes, and cell-type-specific sensitivity to oxidative stress.

Kidneys

Diabetic nephropathy (DN) is one of the most common and severe complications of diabetes, characterized by glomerular hypertrophy, proteinuria, extracellular matrix accumulation, tubulointerstitial fibrosis, impaired waste clearance leading to gradual loss of renal function, and progressive kidney damage [161]. It develops in approximately 30–40% of individuals with either T1D or T2D and remains the leading cause of diabetes-related mortality worldwide [162,163,164]. Ferroptosis has been increasingly recognized as a key contributor to DN progression, primarily affecting nephron cells such as glomerular endothelial cells, podocytes, mesangial cells, and tubular epithelial cells [27,46,165,166,167]. In DN, ferroptosis-related mechanisms may vary across renal cell types but converge on oxidative stress and impaired antioxidant defenses. In tubular epithelial cells and podocytes, exposure to high glucose or ferroptosis inducers leads to ferroptosis, largely through increased ACSL4 and reduced GPX4 and xCT expression [45,168]. Single-cell transcriptomic data identify ceruloplasmin as a regulator of proximal tubular ferroptosis in db/db mice, affecting tissue repair and renin–angiotensin system activity [169,170,171]. Mesangial cells exhibit ferroptosis mediated by HMGB1-driven oxidative stress and S1R-dependent modulation of iron metabolism [168]. Several therapeutic approaches, including Nrf2 activators, sodium-glucose cotransporter 2 (SGLT2) inhibitors, and natural compounds, have demonstrated ferroptosis-inhibitory effects and renoprotective potential in vitro and in diabetic mouse models [24,172,173].

Heart

Diabetic cardiomyopathy (DCM) is a heart muscle disease in diabetic patients without coronary artery disease, hypertension, or obesity, characterized by structural and functional myocardial abnormalities, including diastolic and systolic dysfunction, left ventricular hypertrophy, myocyte hypertrophy, and fibrosis [174,175,176]. It is a major cause of heart failure in patients with diabetes [8]. Recent studies have highlighted the important role of ferroptosis in the pathogenesis of DCM [18,177,178,179]. In db/db and STZ-induced T1D mice, reduced GPX4 and FTH1, along with increased MDA, ACSL4, 4-HNE, and iron content, indicate significant involvement of ferroptosis [180,181,182]. Similar changes, which were attenuated by ferrostatin-1, were observed in H9c2 cardiomyocytes under high-glucose/high-fat conditions [183]. Transcriptome analyses of diabetic mouse hearts confirmed enrichment of ferroptosis-related pathways [177], while isolated cardiac endothelial cells from T2DM mice showed parallel alterations in iron metabolism and lipid peroxidation [184]. Several specific regulatory molecules, detailed in Table 1, modulate ferroptosis in DCM experimental models and link it with inflammation, mitochondrial dysfunction, and endothelial injury [177,184,185,186,187,188,189,190]. Hyperglycemia-induced p53 activation, also detected in vascular tissue, further increases ferroptosis sensitivity via SLC7A11 suppression [191,192,193]. NCOA4-mediated ferritinophagy has also been observed in cardiomyocytes under diabetic conditions [194], and in db/db mice [195] and high-fat diet-fed mice [196]. In addition to cardiomyocytes, cardiac microvascular endothelial cells also undergo pro-ferroptotic alterations [197]. MAPK4 regulates ferroptosis in diabetic cardiac microvascular endothelial cells by suppressing GPX4, reducing dynamin-related protein 1 (Drp1) S-nitrosylation, and mitochondrial translocation. Together, these mechanisms promote myocardial injury, microvascular impairment, and ultimately contractile dysfunction [198]. Therapeutic agents such as sulforaphane, 6-gingerol, H_2_S, isorhapontigenin, baicalin, capsaicin, retinoic acid and irisin alleviate ferroptosis-related myocardial injury by targeting Nrf2-dependent or mitochondrial pathways in diabetic models, both in vitro and in vivo [47,179,182,188,190,199].

Liver

Liver injury significantly contributes to morbidity in both types of diabetes, and is especially prominent in T2D. This is evident not only in altered serum biochemical parameters but also in marked morphological and ultrastructural alterations within liver tissue, including fibrosis, altered proliferation, hypertrophy, and hepatocyte death [27,200,201,202,203]. Our studies on STZ-T1D-induced mice revealed activation of pro-ferroptotic pathways in diabetic rat liver [21], characterized by increased pro-oxidative and pro-inflammatory markers and decreased antioxidative defense. Treatment with Fer-1 reduced these changes and subsequent liver injury, normalized metabolic markers (ALT, triglycerides), and reduced hepatic fibrosis. Similarly, Song et al. (2022) [204] reported ferroptosis-related events in the livers of db/db mice and in HepG2 cells exposed to high glucose. We further found that sulforaphane mitigated hepatic ferroptosis by activating Nrf2, offering a potential therapeutic avenue for diabetes-related liver damage [22]. In addition, recent research has shown that the transcription factor zinc fingers and homeoboxes 2 (ZHX2) also mitigates diabetic liver injury by suppressing ferroptosis [205] through activation of GPX4 transcription, and that in diabetic liver, ferroptosis drives hepatic insulin resistance via hepatocyte injury and mitochondrial dysfunction [206].

Brain

Diabetes increases the risk of several neurodegenerative disorders, including Alzheimer’s disease, Parkinson’s disease, Huntington’s disease, amyotrophic lateral sclerosis, and Friedreich’s ataxia [207]. It also induces cognitive dysfunction, a neurological complication associated with diabetes [208,209]. The brain is particularly vulnerable to iron-dependent oxidative injury, and ferroptosis has been linked to cognitive decline in diabetic encephalopathy. Hippocampal neurons exhibit ferroptotic damage accompanied by memory impairment in experimental diabetes, both in STZ-induced T1D rats and high-fat/STZ-induced T2D mice. These models demonstrate key ferroptotic events associated with memory impairment [210,211,212,213]. Astrocytes and microglia also undergo ferroptosis under hyperglycemia. Reported regulatory pathways of ferroptosis in both neurons and glial cells include FPN1-mediated iron export, AMP-activated protein kinase (AMPK) activation, ER stress, and Nrf2 signaling [210,211,212,213,214,215,216]. Pharmacological and natural agents capable of attenuating these processes demonstrate neuroprotective benefits in diabetic animals and preclinical studies [217,218,219].

Bones

Recent findings indicate that both types of diabetes are frequently associated with impaired bone metabolism, which can lead to osteoporosis and progressive bone deterioration [220]. As a result, diabetic patients, especially elderly individuals, face a significantly increased risk of osteoporotic fractures, negatively affecting their quality of life. These skeletal complications of diabetes, particularly diabetic osteoporosis, also involve ferroptotic mechanisms. Both in vivo and in vitro studies show that bone marrow mesenchymal stem cells experience intensified oxidative stress and ferroptotic signaling (elevated ROS and ACSL4 levels, decreased GPX4 expression, and increased ER stress) under diabetic conditions, impairing their osteogenic potential and differentiation [221,222,223]. This disruption of bone formation contributes to decreased bone quality and increased fracture risk. Natural ferroptosis inhibitors and Nrf2 activators have shown protective effects in preclinical models [222].

Eyes

Diabetic retinopathy (DR) is a common microvascular complication of diabetes and one of the most serious diabetes-related complications, affecting millions of working-age adults globally and representing a leading cause of vision loss worldwide [224]. Its development is primarily driven by retinal microvascular damage, inflammation, and neurodegeneration. In diabetic retinopathy, ferroptosis contributes to both vascular dysfunction and neural degeneration. DR involves ferroptotic disruption of the blood–retinal barrier and neurovascular degeneration [225]. A unique mechanism includes AlkB homologue 5-mediated m6A RNA demethylation, which leads to enhanced YTH N6-methyladenosine RNA binding protein 1-dependent translation of ACSL4. Additional regulatory pathways, including the Flotillin-1/Nrf2 axis and peroxisome proliferator–activated receptor γ (PPARγ) signaling, further define the ferroptotic background of the diabetic retina [226,227,228,229,230,231,232,233].

Skin

Diabetes affects the skin by impairing wound healing, increasing susceptibility to infections, causing diabetic dermopathy, and xerosis. Beyond diminishing quality of life, these complications can predispose patients to more severe outcomes, such as chronic ulcers or infections. Ferroptosis has been implicated in diabetic skin complications, particularly in delayed wound healing in both keratinocytes and fibroblasts [234,235,236,237,238,239]. Evidence of ferroptosis involvement has been reported in wounds in diabetic rat models, where histone lysine crotonylation was shown to accelerate ACSL4-dependent ferroptosis in keratinocytes by modulating autophagy, thereby contributing to impaired wound repair [234]. Targeting ferroptosis, including activation of Nrf2 signaling and administration of natural antioxidants, has been shown to improve wound closure and tissue recovery in diabetic animal models [234,235,236,237,238,239]. Ferroptosis also contributes to diabetes-related limb ischemia in STZ-induced T1D mice and diabetic human endothelial cells; overexpression of aurora kinase A reduces ischemic injury by inhibiting ferroptosis both in vitro and in vivo [239].

Male reproductive system

Impaired spermatogenesis and erectile dysfunction are well-established complications of diabetes mellitus, and growing evidence indicates that ferroptosis is a major mediator of testicular injury in this context [240,241]. This has been demonstrated in STZ-induced T1D mice and high glucose-treated GC-2 testicular cells, where bromodomain-containing protein 7 promoted ferroptotic cell death by enhancing hypermethylation of the clusterin promoter in an enhancer of zeste homolog 2-dependent manner, thereby suppressing AMPK signaling and exacerbating diabetes-related testicular injury [242]. Molecular interventions targeting ferroptosis partly restore reproductive function [243].

Adipose tissue

Adipose tissue dysfunction, particularly visceral fat expansion, drives insulin resistance and T2D, creating a “vicious cycle” in which hyperglycemia and insulin resistance further damage adipose tissue. Metabolic stress in obesity and diabetes promotes conditions that favor ferroptosis in adipose tissue, including iron accumulation, decreased antioxidant capacity, and increased ROS, which enhance lipid peroxidation and affect multiple cell types, including immune cells and neural components involved in metabolic regulation [244]. Hyperglycemia further exacerbates ferroptotic processes by altering heme and iron metabolism, as evidenced by the correlation between the heme exporter FLVCR1 expression and fasting glucose in T2DM patients [245]. Iron dysregulation disrupts adipocyte differentiation, tissue expansion, lipid metabolism, and adipokine secretion, with markers such as serum ferritin linked to reduced adiponectin and impaired insulin sensitivity [246,247]. Interestingly, controlled activation of ferroptotic signaling in adipocytes—through ACSL4 overexpression, ferritin heavy chain deletion, or low-dose ferroptosis agonists—can reduce lipid accumulation and enhance thermogenesis, indicating context-dependent protective roles [248]. This suggests that ferroptotic pathways in adipose tissue can be context-dependent, contributing both to metabolic dysfunction in T2DM and to protective metabolic remodeling under controlled activation.

Overall, ferroptosis in adipose tissue is a mechanistic link between obesity-related remodeling and diabetes, highlighting it as a potential therapeutic target though further studies are needed to clarify its precise role in T2D.

Together, these organ-specific insights highlight ferroptosis as a unifying but context-dependent mechanism in diabetic complications. Table 1 provides a summary of the key cellular targets, molecular events, and model systems used.

**Table 1:** A summary of organ-specific key cellular targets, molecular events, and model systems associated with ferroptosis-dependent mechanisms in diabetic complications.

Tissue/System	Mechanistic Category & Key Alterations	Functional/Pathological Impact	Model Systems	Key References
Pancreatic β-cells	Iron metabolism dysregulation (↑ iron deposition in islets); lipid peroxidation (↑ ACSL4, ↑ MDA, ↑ 4-HNE); antioxidative defense failure (↓ GPX4, ↓ GSH); impaired Nrf2/SLC7A11 axis; decrease in mitochondrial membrane potential	β-cell loss, insulin deficiency	STZ- and HFD/STZ-induced diabetic mice; db/db mice; INS-1 and MIN6 β-cell lines	[19,249,250,251,252,253]
Kidney	Tubular iron overload (↑ Fe^2+^, ↓ ferritin, ↑ TFR1); lipid peroxidation activation (↑ ACSL4, ↑ MDA, ↓ CPT1A expression); antioxidative defense inactivation (↓ GPX4, ↓ GSH, ↓ FSP1, ↓ SOD); Nrf2/HO-1 dysregulation; podocyte ferroptosis (↓ GPX4, ↑ ROS/iron); endothelial ferroptosis; activated NOX4, AMPK/ACC1 inactivation	Tubular injury, podocyte loss, endothelial dysfunction, fibrosis, DN progression	STZ- and db/db mice; HFD/STZ models; HK-2 cells; podocytes; glomerular endothelial cells; human plasma and transcriptomic datasets	[24,45,167,172,254,255,256,257,258,259,260,261,262,263,264]
Heart	Iron overload (↑ Fe^2+^; ↓ FPN1; feritinophagy); enhanced lipid peroxidation (↑ ACSL4/FACL4; ↑ LPCAT3; ↓ ACOT1); antioxidative enzymes downregulation (↓ ATF4/GPX4; ↓ GSH; ↓ PRDX2); suppressed Nrf2/HO-1 signaling; increased inflammation (IL-1β; IL-6; TNF-α); nuclear/circadian dysregulation (REV-ERBα); decreased MFN2	Cardiomyocyte fibrosis and death; DCM progression; I/R injury; microvascular damage	STZ- and db/db diabetic mice; myocardial I/R models; neonatal rat cardiomyocytes; H9c2 cells	[26,47,179,182,190,199,265,266,267]
Liver	Hepatic iron accumulation (↑ iron; ↓ ferritin); lipid peroxidation (↑ ACSL4; ↑ MDA/4-HNE; NOX4); antioxidative defense failure (↓ GPX4; ↓ GSH); autophagy–ferroptosis crosstalk (impaired ACSL4 degradation); suppressed Nrf2/HO-1 signaling	Oxidative liver injury; hepatocyte fibrosis and death; aggravated liver metabolic dysfunction	STZ- and HFD-induced diabetic mice; primary hepatocytes; HepG2 cells	[21,204,205,268,269]
Brain/cognition	Impaired iron export (↑ TFR1; ↓ FTH; ↓ FPN1); lipid peroxidation (↑ ACSL4; ↑ MDA; ↑ NOX2); antioxidative defense failure (↓ GPX4; ↓ GSH; ↓ SLC7A11); Nrf2/HO-1 pathway suppression; PPARα/SLC7A11 dysregulation	Neuronal loss; astrocyte dysfunction; cognitive decline	db/db and STZ diabetic mice; hippocampal and cortical neurons; astrocytes	[210,211,212,213,270]
Bones	Increased oxidative stress (↑ ROS); activation of ferroptotic signaling (↑ ACSL4; ↓ GPX4); ER stress; suppressed Nrf2	Reduced osteogenic differentiation and bone formation; increased osteoporosis	STZ- and HFD-induced diabetic mice; bone marrow mesenchymal stem cells	[220,221,222,223]
Retina/visual pathway	Iron accumulation along visual pathway; GPX4 loss; lipid peroxidation (↑ 4-HNE;); (NOX2-driven ROS); impaired PPARγ signaling;	Photoreceptor and neuronal damage; retinal degeneration	STZ-induced diabetic mice; retinal cell cultures	[216,226,227,228]
Skin/wound healing	Iron overload in wound tissue; lipid peroxidation (↑ MDA); GPX4 loss	Delayed wound closure; impaired healing	STZ-induced diabetic mice; skin wound models	[234,235,236,237,238,239,271]
Male reproductive system	Testis: Iron overload; lipid peroxidation (↑ MDA; ↑ ACSL4; ↑ 4-HNE); antioxidative defense failure (↓ GPX4; ↓ GSH); mitochondrial cristae shrinkage; Penis: Iron accumulation and lipid peroxidation (↑ MDA; ↑ ACSL4); GPX4 deficiency	Impaired spermatogenesis; reduced sperm count and motility; decreased testosterone synthesis; disruption of blood–testis barrier; infertility; erectile dysfunction	STZ-induced diabetic mice/rats; HG–treated Sertoli or Leydig cell lines (e.g., TM4; TM3); GPX4- or Nrf2-rodent models; cavernosal smooth muscle cells	[240,242,272,273,274,275]
Systemic/human evidence	Elevated iron overload markers (↑ ferritin; transferrin saturation); circulating ferroptosis markers (↓ GPX4; ↑ ACSL4; ↑ MDA; ↑ ROS); ferroptosis-enriched transcriptomic signatures	Increased risk of diabetes; DN; ESRD; conserved ferroptosis pathways across species	Human cohorts; plasma biomarker studies; bulk and single-cell transcriptomics	[261,274,276]

Abbreviations used: Acyl-CoA synthetase long chain family member 4 (ACSL4); malondialdehyde (MDA); 4-hydroxynonenal (4-HNE); ferroportin 1 (FPN1); glutathione (GSH); nuclear factor erythroid 2–related factor 2 (Nrf2); solute carrier family 7 member 11 (SLC7A11); fatty acid–CoA ligase 4 (FACL4); lysophosphatidylcholine acyltransferase 3 (LPCAT3); acyl-CoA thioesterase 1 (ACOT1); activating transcription factor 4 (ATF4); glutathione peroxidase 4 (GPX4); peroxiredoxin 2 (PRDX2); heme oxygenase-1 (HO-1); interleukin (IL); tumor necrosis factor (TNF); transferrin receptor 1 (TFR1); mitofusin (MFN); carnitine palmitoyltransferase 1A (CPT1A); ferroptosis suppressor protein 1 (FSP1); superoxide dismutase (SOD); reactive oxygen species (ROS); NADPH oxidase (NOX); AMP-activated protein kinase (AMPK); acetyl-CoA carboxylase 1 (ACC1); endoplasmic reticulum (ER); peroxisome proliferator-activated receptor (PPAR); streptozotocin (STZ); high glucose (HG); high-fat diet (HFD); diabetic nephropathy (DN); diabetic cardiomiopathy (DCM); end-stage renal disease (ESRD).

## 5. Ferroptosis Inhibitors

Ferroptosis inhibitors can be classified by their mechanisms of action as follows: (i) lipid peroxidation inhibitors, which prevent ferroptosis by directly scavenging lipid peroxyl radicals and halting the lipid peroxidation chain reaction; (ii) iron chelators and modulators of iron metabolism; (iii) activators of antioxidative pathways; and (iv) inhibitors of plasma membrane rupture.

(i) Lipid peroxidation inhibitors primarily include radical-trapping antioxidants (RTAs). These may be either lipophilic RTAs that localize to the plasma membrane or RTAs that function within the cytosol. Lipophilic RTAs include synthetic agents such as Fer-1, liproxstatin-1 (Lip-1), α-tocopherol, phenothiazine derivatives, and nitroxides [277,278,279], and endogenous antioxidants such as vitamin E, melatonin, and vitamin K [279,280]. Lipophilicity and membrane localization are essential for their activity. Recent modifications of the diarylamine scaffold of Fer-1 and Lip-1 have significantly improved their potency and metabolic stability. The cholesterol precursor 7-dehydrocholesterol (7-DHC) also functions as a lipophilic RTA, and its accumulation has been shown to protect against ferroptosis in multiple models [279,281]. In the cytosol, several synthetic active compounds such as sulfonamide-phenothiazines, bisbenzylisoquinolines, and hybrid diarylamines can exert potent antiferroptotic effects through RTA activity [282,283,284]. Notably, several clinically approved drugs, including omeprazole, rifampicin, promethazine, carvedilol, and propranolol, have demonstrated RTA-based ferroptosis inhibition and may be suitable for therapeutic repurposing due to favorable pharmacokinetic properties [285]. Additionally, LOX inhibitors (e.g., zileuton) suppress enzymatic peroxidation of PUFAs, while ACSL4 inhibitors (e.g., rosiglitazone) reduce PUFA incorporation into membranes, thereby limiting oxidizable substrates [275,276]. However, ACSL4 regulation in adipose tissue differs from that in other tissues, particularly in the context of lipid remodeling and adipocyte differentiation [286]. Consequently, these regulatory distinctions may influence ferroptosis susceptibility in adipose tissue.

(ii) Iron chelators and iron metabolism modulators, such as deferoxamine (DFO), deferiprone and dexrazoxane, act by reducing the labile iron pool, thereby preventing lipid ROS formation. Early studies showed that the iron chelator, DFO, protects cells from erastin-induced death, while adding free iron enhances it [279,287]. However, iron chelation can be limited, as Fenton reactions can occur in cellular compartments that chelators do not effectively penetrate (e.g., mitochondria) [288]. While protective effects have been observed in some disease models (e.g., traumatic brain injury) [289], clinical outcomes in acute kidney injury have been inconclusive, likely due to poor tissue penetration and pharmacodynamic constraints [290]. In addition to iron chelation, inhibiting ferritinophagy (e.g., NCOA4-targeting agents) or reducing TFR1 expression can also reduce iron availability [291,292]. Many natural antioxidants, such as baicalein, quercetin, berberine, and gallate esters, have potent antiferroptotic effects through multiple mechanisms, including both iron chelation and RTA activity [293,294].

(iii) The third group includes activators of the antioxidative pathway. Compounds that boost GPX4 activity or promote GSH synthesis enhance the detoxification of lipid peroxides and increase resistance to ferroptosis [295,296,297]. Approaches targeting pathways such as the FSP-coenzyme Q10 system, the GTP cyclohydrolase 1 (GCH1)-tetrahydrobiopterin (BH4) antioxidative axis, and pharmacological activation of Nrf2 also strengthen cellular antioxidant capacity and suppress ferroptosis. Agents such as edaravone and dopamine have demonstrated antiferroptotic effects by contributing to cellular antioxidative capacity [298,299].

(iv) The final mechanistic class comprises inhibitors of plasma membrane rupture, currently represented primarily by ninjurin-1 (NINJ1) oligomerization inhibitors. NINJ1 mediates the final step of membrane rupture in ferroptosis, necroptosis, and pyroptosis [300]. Its inhibition or genetic deletion delays membrane rupture and reduces downstream inflammatory responses resulting from cell lysis. This class is distinct in that it targets the execution phase of ferroptosis rather than upstream oxidative reactions. Several novel chemical scaffolds and natural products have shown antiferroptotic activity with potential for translation into therapeutic strategies for diseases such as diabetes. Among these are reactive sulfur metabolites derived from H_2_S (RSS), such as polysulfides and low-molecular-weight persulfides, and H_2_S itself. Their antiferroptotic action is complex, including direct RTA activity and interference with major ferroptotic actors and executors. These mechanisms will be discussed in detail in the following section.

## 6. H_2_S and Protein Persulfidation

Redox signaling is essential for maintaining cellular homeostasis, coordinating responses to environmental stimuli, and mediating defense mechanisms against oxidative stress. Among the three known gasotransmitters—nitric oxide (NO), carbon monoxide (CO), and H_2_S—H_2_S was discovered most recently, opening a new field of H_2_S signaling. The functions of H_2_S and its metabolites have become an area of intense research interest, establishing H_2_S as a key signaling molecule involved in regulating numerous biological processes, including vascular homeostasis and blood pressure [301,302,303], angiogenesis [303,304,305], hypoxia sensing [306,307], inflammation [308], glucose metabolism and insulin secretion [309,310], neurotransmission and neurodegeneration [311,312,313], cell survival, autophagy, and apoptosis [52,314,315], and aging [316,317]. The biochemical properties and (patho)physiological functions of H_2_S have been comprehensively reviewed elsewhere [33,51,318,319] and will not be discussed here in detail. In this section, we focus on the signaling pathways of H_2_S in diabetes-targeted tissues and the impact of their (dys)regulation.

### 6.1. H_2_S Synthesis and Signaling Pathways

**Figure 3:**
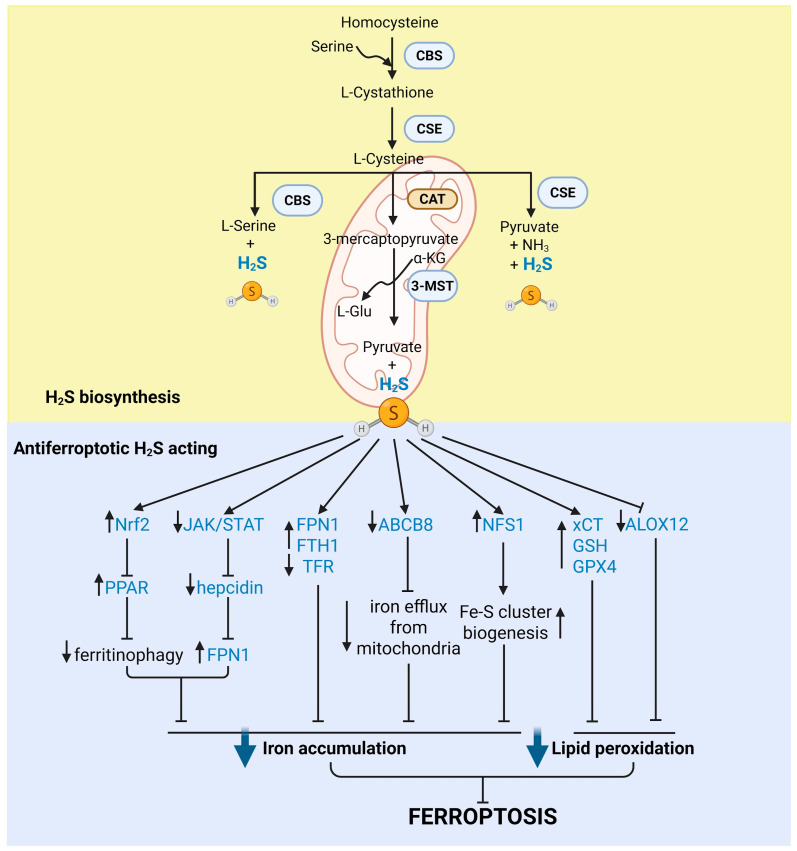
Biosynthesis of hydrogen sulfide (H_2_S) and its antiferroptotic signaling. H_2_S is produced endogenously through enzymatic pathways involving three key enzymes: cystathionine β-synthase (CBS), cystathionine γ-lyase (CSE), and 3-mercaptopyruvate sulfurtransferase (3-MST). CBS and CSE function in the transsulfuration pathway, where CBS converts homocysteine to cystathionine, and CSE subsequently converts cystathionine to L-cysteine. L-cysteine then serves as a substrate for H_2_S production by both CBS and CSE, with CBS generating L-serine and CSE producing pyruvate and ammonium (NH_3_) as by-products. In addition, predominantly in mitochondria, 3-MST produces H_2_S from 3-mercaptopyruvate, which is generated from L-cysteine by cysteine aminotransferase (CAT), linking H_2_S synthesis to mitochondrial sulfur metabolism. Reactive sulfur metabolites derived from H_2_S (RSS) activate nuclear factor erythroid 2-related factor 2 (Nrf2), which, via peroxisome proliferator-activated receptor (PPAR) signaling, suppresses ferritinophagy. RSS also inhibits the Janus kinase/signal transducer and activator of transcription (JAK/STAT) pathway, reducing hepcidin synthesis and increasing ferroportin (FPN1) expression. RSS further increases FPN1 and ferritin (FTH1) levels while decreasing transferrin receptor 1 (TFR1) expression, leading to reduced intracellular free iron. Moreover, RSS limits iron efflux from mitochondria by downregulating the mitochondrial iron transporter ATP-binding cassette subfamily B member 8 (ABCB8), thereby lowering cytosolic free iron. RSS also increases expression of NFS1 cysteine desulfurase (NFS1), enhancing the biogenesis of Fe-S clusters. Beyond iron regulation, RSS decreases lipid peroxidation by enhancing antioxidant defenses through upregulation of the cystine/glutamate antiporter system (xCT), glutathione peroxidase 4 (GPX4), and glutathione (GSH), and by suppressing arachidonate 12-lipoxygenase (ALOX12)-mediated polyunsaturated fatty acid (PUFA) lipid peroxidation. Created in BioRender. Vidakovic, M. (2026) https://BioRender.com/qlvom2y (accessed on 29 December 2025).

H_2_S is produced endogenously through enzymatic pathways that primarily involve three key enzymes: cystathionine β-synthase (CBS), cystathionine γ-lyase (CSE), and 3-mercaptopyruvate sulfurtransferase (3-MST) (Figure 3).

CBS and CSE participate in the transsulfuration pathway, an enzymatic process in which homocysteine transfers sulfur to cysteine [320]. Recently, an enzymatic pathway for H_2_S production that involves human selenium-binding protein 1–a methanethiol oxidase–has also been identified in the intestines [321]. In addition to their presence in the cytoplasm, CBS and CSE may be found in other cellular compartments, such as the nucleus or mitochondria. In contrast, 3-MST is predominantly located in the mitochondria. Although all three enzymes are involved in H_2_S generation, they display tissue-specific expression: in the central nervous system, CBS is expressed in astrocytes and glial cells, whereas CSE is predominantly found in neurons; CSE is abundantly expressed in the vascular endothelium and liver, and 3-MST is widely distributed throughout the body [320]. The synthesis of H_2_S is tightly regulated and influenced by various (patho)physiological factors, including oxidative stress, inflammatory cytokines, and hormonal signals [51,319,322], allowing it to function as a dynamic signaling molecule in tissue homeostasis and disease progression. As a versatile redox mediator, H_2_S influences numerous cellular processes through three principal mechanisms: (i) binding to metal centers in metalloproteins, and engaging in subsequent redox chemistry; (ii) reacting with and neutralizing ROS and RNS; and (iii) post-translational modification of protein thiols through persulfidation (S-sulfhydration) [51]. A key feature of H_2_S chemistry is its dual role as a reducing agent and a metal ligand. At physiological pH, H_2_S predominantly exists as the hydrosulfide anion (HS^−^), which can directly bind to transition metal centers in metalloproteins, altering their redox state and function. For example, in cytochrome c oxidase, H_2_S acts as an electron donor at low concentrations, transiently enhancing respiration, whereas at higher levels it binds to the enzyme to form stable metal-sulfide complexes that inhibit oxygen binding and electron transfer [323]. In hemoglobin and myoglobin, H_2_S binds to ferric heme iron, forming Fe(III)–HS^−^ adducts that can be oxidized to RSS, such as polysulfides and thiosulfate [324,325], contributing to the cellular persulfide pool. These interactions highlight the integration between heme protein chemistry, persulfidation-based signaling and antioxidative defense.

H_2_S also interacts extensively with ROS and RNS. Reactions with NO produce hybrid species, such as thionitrous acid (HSNO), which can act as transnitrosating agents, or nitroxyl (HNO), a redox-active signaling molecule in its own right [326]. These reactions exemplify the chemical coupling between sulfide and nitrogen signaling pathways and highlight the ability of H_2_S to serve both as a scavenger of oxidants and as a generator of novel RSS with distinct biological functions. Furthermore, H_2_S can neutralize superoxide, H_2_O_2_, hypochlorous acid, and peroxynitrite [51,327,328,329], while being converted to oxidized sulfur intermediates such as persulfides and polysulfides [330]. These reactive metabolites of H_2_S, generated through interplay with ROS and RNS, are mechanistically linked to protein persulfidation and are crucial for the central post-translational modification underlying H_2_S signaling, persulfidation [51].

Persulfidation, also known as S-sulfhydration, is an evolutionarily conserved oxidative posttranslational modification in which thiol groups in cysteine residues are converted into persulfides. This reversible, redox-based posttranslational modification is a targeted mechanism by which H_2_S fine-tunes protein function and cellular responses [51,331]. Compared to the first two modes of H_2_S action, persulfidation is now recognized as the central mechanism underlying H_2_S-mediated biological effects [51,317,319,331,332,333]. Persulfidation can occur through multiple mechanisms: (1) direct reaction of H_2_S with oxidized thiols such as sulfenic acids (–SOH); (2) trans-persulfidation involving intermediate sulfur donors; or (3) reaction with polysulfides generated from H_2_S oxidation [51]. The efficiency of persulfidation depends on the redox state of the target protein, the local concentration of H_2_S, and the presence of catalytic cofactors or transition metals. This crucial, evolutionarily conserved mechanism is reversible, with a strong regulatory loop. Depersulfidation is catalyzed by the GSH/glutaredoxin and thioredoxin-reducing systems [334,335,336].

Persulfidation serves as an adaptive modification that protects cysteine residues from over-oxidation by ROS and preserves thiol functionality [331,333,337]. Under oxidative stress, cysteine thiols (P–SH) can be irreversibly oxidized to sulfonic acid (P–SO_3_H) through a sulfinic acid (P–SO_2_H) intermediate, often resulting in loss of protein function [338,339]. In contrast, persulfidated cysteines (P–SSH) can be oxidized by ROS to form P–S–SO_3_H, which is readily reduced back to P–SH by GR and TR [317,331,333,337]. By altering the chemical reactivity of cysteine residues, persulfidation modulates enzyme activity, protein–protein interactions, and protein stability. For example, persulfidated glycolytic enzyme glycerol-3-phosphate dehydrogenase (GPDH) alters its catalytic activity in various model organisms [340]. MnSOD can also be persulfidated, preserving its activity and increasing its resilience to tyrosine nitration by peroxynitrite [317]. Persulfidation rescues neuronal survival, improves cognition in Alzheimer’s disease models, and protects DJ-1 and Parkin function in Parkinson’s disease [51,333,341].

Persulfidation is critical in redox-regulated signaling cascades. In the classical Keap1-Nrf2 pathway, persulfidation of Keap1 cysteine residues induces conformational changes that release Nrf2, allowing it to translocate to the nucleus and initiate the expression of antioxidative genes [342]. This links sulfide metabolism to cellular antioxidative defense, as reported in cardiovascular and neurodegenerative models [57].

Anti-inflammatory and metabolic effects of persulfidation have also been reported. Specifically, persulfidation of Sirtuin 1 (Sirt1) ameliorates inflammation and insulin resistance [343], while NF-κB persulfidation results in its cytoplasmic retention and inhibition of DNA-binding activity [344]. H_2_S also modulates autophagy through persulfidation of autophagy regulators and affects apoptosis-related pathways (e.g., by modifying caspases or B-cell lymphoma 2, BCL-2 family proteins), contributing to either cytoprotection or cell death under different stress conditions [345].

H_2_S modulates ion channel activity directly or via persulfidation. A canonical example is activation of ATP-sensitive K^+^ (K_ATP_) channels in vascular smooth muscle, leading to membrane hyperpolarization and vasodilation. These effects, along with those on other channels (transient receptor potential (TRP) family, voltage-gated channels), explain at least in part the role of H_2_S in blood pressure regulation [346,347].

Persulfidation generally confers cytoprotection under oxidative stress, enhancing cellular resilience and survival. Through reversible modification of cysteine residues, persulfidation provides a finely tuned mechanism for cellular adaptation to stress. Loss of persulfidation capacity is associated with aging, metabolic dysfunction, chronic inflammation, cardiovascular pathology, neurodegeneration and cancer, making H_2_S-based therapeutics (donors, enzyme activity modulators) a promising area of ongoing research [348].

### 6.2. H_2_S and Ferroptosis Regulation

The biological role of H_2_S is closely linked to ferroptosis, as the substrates, enzymes, and donors involved in H_2_S production intersect with those that regulate redox balance and iron homeostasis. Cystine, imported via xCT, is converted to cysteine, which serves as a precursor for GSH and as a substrate for H_2_S production. Thus, metabolic flux through cysteine directly connects H_2_S synthesis to GSH-dependent antioxidant defense.

Many studies have shown that H_2_S can interfere with various ferroptotic targets, leading to decreased iron accumulation and lipid peroxide production, ultimately suppressing ferroptosis. Inhibition of ferroptosis by H_2_S involves several metabolic and signaling pathways, including iron metabolism, lipid metabolism, and antioxidative defense. In iron metabolism, it has been repeatedly shown that administration of H_2_S donors can increase the expression of FTH1 and FPN while reducing the expression of TFR [349,350], all of which decrease free iron in the cell. The effects on FPN can include regulation of hepcidin, as H_2_S donors can reduce hepcidin expression via inhibition of the JAK–STAT pathway, thereby preventing hepcidin-mediated FPN1 downregulation and consequent intracellular iron accumulation [351]. Furthermore, activation of the Nrf2/PPAR axis by H_2_S administration can suppress ferritinophagy, thereby decreasing intracellular iron [352]. Additionally, H_2_S decreases the expression of mitochondrial ATP-binding cassette subfamily B member 8 (ABCB8) protein, resulting in reduced iron efflux from mitochondria [352]. The anti-ferroptotic effect of H_2_S can also be achieved through stabilization of Fe-S cluster–containing proteins [352].

A broad range of H_2_S anti-ferroptotic actions is described in the context of enhancing antioxidative defense and suppressing lipid peroxidation. Specifically, exogenous H_2_S donors, such as sodium hydrosulfide (NaHS), can increase GSH levels and enhance GPX4 activity, suppressing ferroptosis by reducing lipid peroxidation [348]. H_2_S donors may also stabilize xCT through persulfidation of Otubain-1 (OTUB1), a deubiquitinase that protects xCT from proteasomal degradation, which could affect the xCT/GSH/GPX4 ferroptosis axis [353]. Additionally, H_2_S can interfere with enzymatic production of lipid peroxides, as NaHS inhibits both the expression and acetylation of arachidonate 12-lipoxygenase (ALOX12), a key enzyme that catalyzes PUFA–phospholipid peroxidation, thereby protecting myoblasts from ALOX12-dependent ferroptotic cell death [354]. Some reactive sulfur species, specifically hydropersulfides, can also inhibit ferroptosis by directly removing phospholipid-derived peroxyl radicals [342]. Since persulfidation is recognized as a common mechanism of H_2_S bioactivity, it remains to be explored whether the effects of H_2_S on ferroptosis-related parameters involve this direct interaction of its reactive species with target proteins. To date, such a mechanism has been reported for the regulation of xCT and Nrf2, specifically their regulatory protein OTUB1, as mentioned above, and Keap-1, respectively [47,342,353].

Due to the complex, pleiotropic interactions of H_2_S with ferroptosis-related signaling pathways, targeted inhibition of ferroptosis using H_2_S donors could be a promising therapy for many pathological states involving ferroptosis as a pathological mechanism [347,348,352,354,355]. Many diseases, such as chronic obstructive pulmonary disease [356], myocardial diseases [354,357], endothelial dysfunction in atherosclerosis [342], and ischemic stroke brain injury [357], have recently been found to be alleviated directly by H_2_S inhibition of ferroptosis. These studies were mainly conducted in cell or animal models of the diseases, and clinical data specifically describing the effects of H_2_S on ferroptosis are still lacking. In general, for the clinical application of H_2_S donors, there is an ongoing effort to find donors that are safe, effective, and tolerable in clinical settings. To the best of our knowledge, only two H_2_S donor-based therapies, a H_2_S-releasing antiinflammatory and analgesic drug, ATB-346, and a synthetic H_2_S precursor, SG1002-have been tested and confirmed as beneficial in phase I and II clinical trials [358,359]. Interestingly, a large number of known drugs and molecules have been shown to release H_2_S, which brings hope for their repurposing in therapeutic applications, including those based on ferroptosis inhibition. All of these factors may participate in the reduction in ferroptosis in diabetes and associated pathologies.

## 7. Disturbances of H_2_S Signaling in Diabetes

The relationship between H_2_S levels and diabetes is an emerging area of research that highlights the importance of this gasotransmitter in metabolic regulation and diabetic complications. Altered H_2_S production and regulation have been demonstrated in diabetes, suggesting that dysregulation of H_2_S may contribute to β-cell dysfunction, insulin resistance, and target-organ injury.

Early studies showed that glucose stimulation increases H_2_S production in pancreatic islet cells [360], where H_2_S acts as a negative regulator of insulin secretion by activating K_ATP_ channels, reducing ATP levels, and modulating Ca^2+^ signaling [361,362,363]. In vitro, H_2_S or CSE overexpression induces ER stress and apoptosis in INS-1E cells via the p38 MAP kinase pathway [364], while CSE inhibition prevents STZ-induced cell death, indicating a pro-apoptotic role for endogenous H_2_S under certain conditions [363]. Several groups have shown that compounds suppressing H_2_S synthesis enhance insulin secretion [363,365], supporting the hypothesis that increased H_2_S production in diabetic islets contributes to β-cell dysfunction during diabetes progression [50].

However, other studies suggest that the rise in H_2_S during early hyperglycemia serves as an adaptive cytoprotective mechanism, functioning as an “intrinsic, pancreatic brake” to prevent β-cell exhaustion [366]. H_2_S has been shown to preserve β-cell viability and insulin secretion in mouse islets and MIN6 cells exposed to diabetogenic conditions [365,366]. Data from in vitro studies clearly show that the role of H_2_S in β-cell function and insulin secretion is complex, highly context- and dose-dependent, and varies with disease stage and experimental model [367].

Conflicting outcomes are also observed in vivo. Increased CBS expression has been reported in STZ-treated diabetic animals [355], and CSE inhibition improved glycemic control in diabetic models [362,367]. Moreover, CSE knockout delays diabetes onset and preserves β-cell mass [367]. Conversely, mice fed a high-fat diet and deficient in CSE exhibit worsened islet glucotoxicity compared to wild-type counterparts, highlighting the dual and dynamic role of H_2_S [368]. Cytoprotection by H_2_S is partially mediated through TRX activation, which maintains redox balance and protects β-cells against glucotoxic stress. The growing body of research examining the dual actions of H_2_S in the pancreas increasingly supports its predominantly protective role. Recent in vivo findings indicate that H_2_S confers beneficial effects not only on pancreatic function but also on peripheral tissues involved in glucose homeostasis [309,369,370,371,372,373,374].

H_2_S has emerged as a regulator of a pathological hallmark of T2D–insulin sensitivity–in the liver, adipose tissue, and skeletal muscle. The impact of diabetes on hepatic H_2_S production remains controversial. Some studies report increased H_2_S production and elevated CSE/CBS expression in the liver of STZ-diabetic rats, with these changes reversed by insulin treatment [371]. In contrast, other studies have found decreased H_2_S formation and CSE activity in the same model [372]. Thus, the role of H_2_S in the diabetic liver appears to depend profoundly on the type and stage of diabetes, the experimental model used (in vitro vs. in vivo), and the signaling pathways involved. In adipose tissue, H_2_S also exerts context-dependent effects on glucose metabolism. On one hand, H_2_S and RSS can promote insulin sensitivity and lipid storage. For example, they: (i) enhance insulin responsiveness under high-glucose conditions in 3T3-L1 adipocytes by boosting phosphatidylinositol 3,4,5-trisphosphate levels; (ii) improve insulin signaling by activating insulin receptors in insulin-resistant diabetic rats; (iii) facilitate glucose conversion to triglycerides via PPARγ activation; and (iv) promote adipogenesis by increasing expression of fatty acid-binding protein 4 [309,373,374]. On the other hand, data indicate that H_2_S can contribute to insulin resistance in fat cells, particularly in response to inflammatory signals such as tumor necrosis factor α TNF-α [373]. In skeletal muscle, H_2_S appears to exert predominantly beneficial effects by improving insulin sensitivity through the insulin receptor-phosphatidylinositol 3-kinase-protein kinase B (IR–PI3K–Akt) signaling cascade. In C2C12 myoblasts, treatment with NaHS increases glucose uptake, supporting a positive role for H_2_S in muscle glucose utilization [369,370].

Clinical and experimental evidence indicate that systemic H_2_S availability is diminished in diabetes. Patients with T2D have significantly reduced plasma H_2_S levels, which correlate with poor glycemic control as indicated by elevated glycated hemoglobin (HbA1c) levels [36,38]. Similar progressive reductions in circulating H_2_S have been reported in several experimental models, including T1D (non-obese diabetic, NOD mice and STZ-treated rats), and T2D (high-fat diet–induced T2D in rats and db/db mice) [36,375,376,377,378]. The reduced levels of H_2_S in circulation may result from decreased endogenous tissue production under diabetic conditions [378,379]. However, they may also result from rapid local consumption within targeted tissues, where H_2_S is quickly utilized to counteract heightened oxidative stress, despite increased production. Consequently, reduced systemic H_2_S availability exacerbates oxidative stress and inflammation in diabetes-targeted tissues [380], both of which are central drivers of diabetic complications such as cardiovascular disease and DN [380,381].

Endogenous H_2_S deficiency, both systemically and within the kidneys, is increasingly recognized as a contributor to the onset and progression of DN in animal studies [36,382,383,384,385,386,387]. Experimental models of both T1D and T2D demonstrate downregulation of renal CBS and CSE expression [383,387,388,389]. Clinically, reduced plasma H_2_S levels have been observed in diabetic patients undergoing chronic hemodialysis compared with non-diabetic hemodialysis patients [390]. Hyperglycemia-mediated suppression of CSE-dependent H_2_S synthesis is proposed to accelerate the progression of renal injury.

Recent studies also implicate H_2_S deficiency in the development of DCM [391,392]. Reduced H_2_S bioavailability in diabetes promotes oxidative stress, mitochondrial dysfunction, ER stress, necroptosis, and NLR family pyrin domain containing 3 (NLRP3) inflammasome activation, while H_2_S supplementation (e.g., NaHS) reverses these pathological processes and improves cardiac function [393,394,395]. A key role for H_2_S in diabetes-induced vascular endothelial injury has also been reported, but changes in H_2_S-producing enzymes remain inconsistent. Some studies report no alterations in CSE, CBS, or 3-MST expression [41,396], while others show reduced CSE expression and H_2_S levels in diabetic endothelial cells and vessels [397,398,399,400]. Regarding DR, data show altered H_2_S levels: increased in aqueous humor samples [401] but decreased H_2_S-producing enzymes in retinas from patients with proliferative DR [402].

Collectively, these findings highlight the complex interplay between H_2_S levels and diabetes pathophysiology. Altered H_2_S production and bioavailability exacerbate oxidative stress, inflammation, and subsequent tissue injury. Therefore, restoring or modulating H_2_S levels represents a promising therapeutic approach to mitigate diabetes progression and its complications.

## 8. H_2_S and RSS Donors as a Strategy in Diabetes Management

### 8.1. Classification of H_2_S and RSS Donors

H_2_S donors are chemical compounds designed to release H_2_S in a controlled and biologically relevant manner, mimicking or enhancing its endogenous effects. Because H_2_S is a short-lived molecule, direct administration is not feasible; therefore, donor-based strategies are essential for experimental and translational applications [403,404]. Differences in donor chemistry and release kinetics critically influence H_2_S bioavailability and biological effects, underscoring the importance of appropriate donor selection.

Inorganic sulfide salts such as NaHS and sodium sulfide (Na_2_S) release H_2_S immediately upon use, providing a rapid and transient increase in its concentration. These compounds are useful for studying the acute effects of H_2_S but may not accurately reflect physiological H_2_S signaling due to their short half-life and non-specific distribution. To address these limitations, slow-releasing donors have been developed, with GYY4137 (4-methoxyphenyl) (morpholino)phosphinodithioic acid) being the most widely used. It provides sustained H_2_S delivery over several hours, better replicating endogenous H_2_S production [405,406].

Slow-release donors are especially valuable for assessing cytoprotective, anti-inflammatory, and metabolic benefits under chronic or stress conditions. More recently, mitochondria-specific H_2_S donors such as AP39, AP123, and AP1060 have been synthesized to deliver H_2_S directly to mitochondria, the main site of its signaling and redox regulation [407,408]. These donors improve mitochondrial function, reduce oxidative stress, and protect cells from apoptosis and ferroptosis, highlighting their therapeutic potential in cardiovascular, neurodegenerative, and metabolic disorders.

Natural and hybrid donor systems expand the range of pharmacological applications by coupling H_2_S delivery with other therapeutic activities [409]. Natural H_2_S donors include organosulfur compounds found in foods such as garlic and onions (e.g., allicin), and cruciferous vegetables (e.g., isothiocyanates) [410], while hybrid donor systems include H_2_S-releasing derivatives of nonsteroidal anti-inflammatory drugs and carbonyl sulfide-based donors.

Beyond direct H_2_S donors, increasing attention has focused on donors of RSS, such as polysulfides and persulfides, which enhance protein and low molecular weight thiol persulfidation, a key post-translational mechanism of H_2_S signaling [411,412,413].

### 8.2. RSS Donors in Diabetic Complications

The use of H_2_S donors has been recognized as an advanced strategy for managing diabetes and its complications, including nephropathy [380], cardiomyopathy [414], retinopathy [43,415], endothelial dysfunction [416,417], accelerated atherosclerosis [418], wound healing and diabetes-associated cognitive decline [49]. The common mechanisms of action include reducing oxidative stress, inhibiting inflammatory signaling, improving mitochondrial function, inhibiting fibrosis pathways, and reducing cell death, primarily apoptosis. Most effects involve interference by H_2_S with multiple signaling pathways and molecular targets, resulting in improved structure and function of targeted tissues. A detailed literature overview, including the type of diabetes complication, the donor used and the mechanism involved, is presented in Table 2, while the major mechanistic aspects of H_2_S action in various diabetic complications are described below.

The main antioxidative mechanism of H_2_S action, which involves activation of Nrf-2 signaling and regulation of downstream targets such as HO-1 and NAD(P)H quinone dehydrogenase 1 (NQO1), is observed in almost all diabetic complications, including DCM [420,455], DN [436], endothelial dysfunction and atherosclerosis [342]. In DCM, H_2_S has a positive effect on another redox sensor, forkhead box protein O1 (FOXO1), leading to increased phosphorylation [428]. In addition, decreased NOX4-related ROS production with H_2_S donors has been observed in myocardial cells in diabetic rat models [456] and in renal tubular epithelial cells under high glucose conditions [388].

The anti-inflammatory effects of H_2_S in diabetic complications commonly involve NF-kB-related signaling. H_2_S donors prevent diabetes-induced cardiac inflammation by inhibiting JNK/leptin/p38 MAPK/toll-like receptor 4 (TLR4) and NF-κB/NLRP3 signaling pathways [427,457]. Similar effects mediated by NF-κB-induced cytokine inflammation have also been observed in DN [389], atherosclerosis, endothelial dysfunction [444,458] and DR [415]. Additionally, H_2_S donors attenuate inflammation and increase angiogenesis in diabetic wound healing [400].

The mechanisms underlying the antifibrotic effects of H_2_S donors are extensively described in DCM and DN. Studies of DCM have shown that H_2_S affects several pathways that protect against diabetes-induced tissue hypertrophy and fibrosis, including downregulation of the canonical Wnt and transforming growth factor β1 (TGF-β1)/Smad3 signaling pathways [421], suppression of ER stress [391,419,422,459], blockade of the PKC and ERK1/2 pathways [423], inhibition of the JAK/STAT pathway [424], and activation of the PI3K/Akt signaling pathway [425]. In DN, H_2_S reduces global and matrix protein synthesis in high glucose-challenged renal epithelial cells through induction of AMPK phosphorylation and inhibition of mechanistic target of rapamycin kinase (mTORC1) activation [384].

Regarding the improvement of mitochondrial function in diabetic models by H_2_S, comprehensive data are available for DCM and diabetes-related endothelial dysfunction. H_2_S regulates mitochondrial ROS production, enhances mitochondrial respiratory chain activity and ATP production [407,432], and controls the balance between mitochondrial fusion and fission, favoring mitophagy as a protective mechanism that enables removal of damaged mitochondria [398,432], in both diabetic animal models and diabetic-mimicking conditions in vitro. Beyond DCM, positive effects of H_2_S on mitochondrial dysfunction—including ROS production, ATP generation and mitochondrial swelling—have been reported in retinal samples from diabetic rats [43,415].

**Figure 4:**
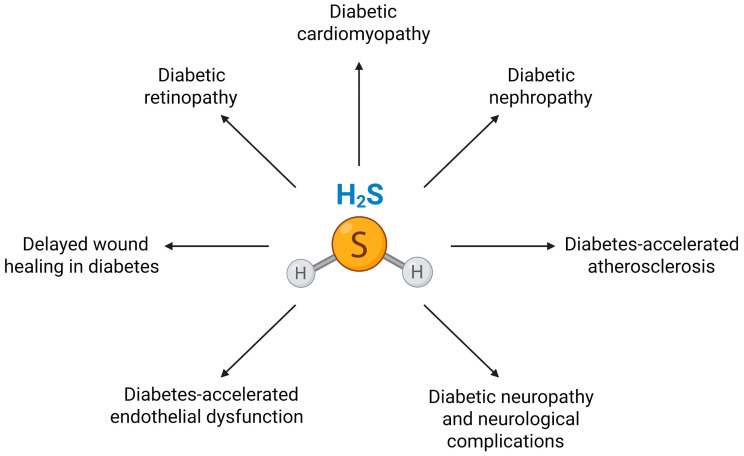
Protective effects of H_2_S in diabetic pathologies. Treatment with H_2_S donors can mitigate diabetic retinopathy, cardiomyopathy, nephropathy, diabetes-accelerated endothelial dysfunction and atherosclerosis, neuropathy and neurological complications, and delayed wound healing. Created in BioRender. Vidakovic, M. (2026) https://BioRender.com/wvbr5n9 (accessed on 29 December 2025).

Equally important are the effects of H_2_S on cell death in diabetic conditions, mainly through the regulation of apoptosis and autophagy. In DN, the antiapoptotic effect of H_2_S on renal tissue of diabetic rats and on high-glucose-induced podocyte apoptosis has been observed, mediated by modulation of the SIRT1/p53 apoptosis pathway. H_2_S also inhibits cardiomyocyte apoptosis by suppressing p38 MAPK/JNK and STAT3/hypoxia-inducible factor 1α (HIF-1α) signaling pathways, alleviating calcium deposition, and activating the PI3K/Akt signaling [420]. PI3K/Akt/endothelial NOS signaling is also a target for H_2_S in diabetes-related apoptosis of endothelial cells [448]. Additionally, H_2_S inhibits apoptosis in retinal microvascular endothelial cells [44], indicating an antiapoptotic role of H_2_S against DR. Induction of autophagy has been reported as a protective mechanism of H_2_S action in DCM [460]. A schematic presentation of the positive effects of H_2_S on diabetic pathologies is shown in Figure 4.

Antiferroptotic effects of H_2_S are recognized as a novel mechanism underlying its antidiabetic actions. To the best of our knowledge, antiferroptotic actions have been reported in high-glucose-induced osteoblast injury in vitro [461], and in animal models of DCM [47] and diabetes-induced anxiety- and depressive-like behaviors [454]. Common mechanisms include positive effects of H_2_S on ferroptosis-related parameters such as lipid peroxide levels, iron, GPX4, and xCT. Specifically, in cardiomyocytes, H_2_S regulates the Nrf2/GPX4/GSH pathway through sulfhydration of the E3 ligase synoviolin (Syvn1), an enzyme that regulates Keap1 ubiquitination and consequently Nrf2 activation.

Regarding the antiferroptotic effects of H_2_S, it is essential to consider the activity of its reactive metabolites, as recent studies have demonstrated that low-molecular-weight persulfides possess strong antiferroptotic potential. Specifically, hydropersulfides have been identified as excellent hydrogen atom transfer agents [462] and have been shown to react efficiently with phospholipid-derived peroxyl radicals, with kinetics comparable to those of the most effective inhibitors of ferroptosis described to date [463]. In this context, it is particularly important to employ compounds or donors capable of selectively enhancing the delivery, bioavailability, and activity of RSS. In addition, since persulfidation—a key mechanism of H_2_S signaling—requires prior oxidation and generation of polysulfides and persulfides, a range of sustained-release donors capable of delivering these oxidized sulfur species has been synthesized and explored in recent years. Considering that sulfide-to-persulfide chemistry is complex, as these species exist in dynamic biological equilibrium, different pharmacological approaches to increase persulfide content must be used.

Most of the above-mentioned studies used inorganic H_2_S salts such as NaHS, slow-releasing donors like GYY4137, or mitochondria-targeted donors such as AP123 and AP39. Accordingly, data on S-sulfhydration as a mechanism of protective action in diabetic complications remain limited. Recently, it was reported that persulfidation of target molecules regulates cardiac structural damage, mitophagy, and lipid droplet accumulation in the hearts of db/db mice treated with NaHS [431,432,464]. Recently published data by Su et al. (2025) show that NaHS improves mitochondrial activity in diabetic hearts through S-sulfhydration of mitochondrial respiratory complexes [465]. However, increased use of polysulfide (Na_2_S_4_) in studies of H_2_S has led to recognition of persulfidation as an important antidiabetic mechanism of H_2_S donors. For example, it has been shown that polysulfide prevents DN via phosphorylation and acetylation of p65 NF-κB and STAT3 mediated by sulfhydration of SIRT1 [466]. Also, Na_2_S_4_ prevented the development of DCM via sulfhydration of both PPARγ and SIRT3 [429].

The antidiabetic effects of persulfide donors have not yet been explored, even though persulfides are biologically relevant sulfur species. Considering their strong antiferroptotic potential, we recently compared the effects of three different H_2_S/RSS donors-the persulfide donor cysteine-trisulfide (Cys-3S), the slow-realizing H_2_S donor GYY4137, and the polysulfide donor Na_2_S_4_ in relation to diabetes development in an animal model. We have shown that the direct persulfide donor Cys-3S exhibits the strongest antidiabetic potential, resulting in a decreased incidence of diabetes and maintenance of a functional β-cell population through suppression of the ferroptotic phenotype [467]. Furthermore, our previous and ongoing work indicates that H_2_S/RSS donors provide significant hepatoprotection during diabetes progression by attenuating ferroptosis-driven liver injury [468]. These findings qualify direct persulfide donors as candidates for diabetes treatment and pave the way for further study of their antidiabetic mechanisms, particularly the process of persulfidation.

## 9. Further Directions

Although considerable progress has been made in defining the role of ferroptosis in the pathogenesis of diabetes and identifying H_2_S as a potential modulator of this process, many questions remain. Future research should prioritize systematic efforts to clarify the dual and context-dependent role of H_2_S in pancreatic β-cell function and survival, as this will provide insight for optimizing therapeutic strategies. Another important direction is to investigate how ferroptosis contributes to diabetes progression across different tissues. The interplay between ferroptosis and other regulated cell death pathways, such as apoptosis, necroptosis, and autophagy, also requires deeper exploration. Unraveling these interconnections will provide a more comprehensive understanding of how redox imbalance and iron overload drive cellular demise in diabetes. In particular, studies focusing on the role of ferroptosis in tissue-specific complications and its temporal dynamics during disease progression may identify new therapeutic opportunities. The development and optimization of H_2_S/RSS donors with improved pharmacokinetic properties, such as enhanced delivery, controlled release, and tissue specificity, represent another promising avenue. Therefore, novel persulfide or polysulfide donors should be more extensively evaluated in both preclinical and clinical settings. In addition, strategies aimed at enhancing protein persulfidation may provide new tools for achieving the antiferroptotic, antioxidative, and anti-inflammatory effects of H_2_S. Research directed toward modulating the persulfidation patterns of key ferroptosis regulators may open new avenues for diabetes treatment. In this regard, systematic screening of donor specificity, release kinetics, and tissue distribution will be essential for defining safe and effective therapeutic protocols.

Another major challenge lies in translating experimental findings into clinical practice. While preclinical models provide strong evidence for H_2_S-mediated protection in diabetic complications, human studies remain limited. Well-designed clinical trials are needed to evaluate the safety, efficacy, and optimal dosing of H_2_S donors or persulfidation-enhancing agents in diabetic populations. To facilitate this, biomarkers of ferroptosis and persulfidation should be developed and validated to enable early detection of ferroptotic processes in patients and to monitor therapeutic responses. Combining such biomarkers with advanced imaging and multi-omics approaches could help identify individuals most likely to benefit from targeted H_2_S/RSS-based interventions.

Finally, the broader metabolic context of H_2_S and ferroptosis warrants further exploration. Interactions with gut microbiota, dietary sulfur sources, and systemic iron metabolism may profoundly influence ferroptotic susceptibility and H_2_S bioavailability in diabetes. Integrative studies combining molecular biology, pharmacology, and systems-level approaches are needed to capture this complexity. Moreover, comparative studies across T1D and T2D, and between early and late disease stages, may uncover disease-specific vulnerabilities that can be therapeutically exploited.

Overall, these research directions hold great promise for advancing innovative approaches that target the root causes of diabetes and its complications by modulating ferroptosis through H_2_S signaling. Such efforts will be essential for translating the concept of ferroptosis modulation by H_2_S into effective prophylactic and therapeutic strategies.

## Figures and Tables

**Table 2:** The effects of H_2_S and RSS donors on diabetic complications.

Pathology	H_2_S Donor	Mechanisms of Action	Main Beneficial Effects	Key References
Diabetic cardiomyopathy (DCM)	NaHS, GYY4137, Na_2_S_4_	Activation of Nrf2 → HO-1, NQO1, inhibition of p38-MAPK/JNK, PKC/ERK1/2, JAK/STAT, Wnt/WISP-1 and TGF-β1/Smad3, and activation of PI3K/Akt; suppression of NF-κB/TLR4/NLRP3-mediated inflammation and pyroptosis; preservation of mitochondrial function; activation of Nrf2/GPx4/GSH pathway; acts via protein persulfidation	Improved systolic and diastolic cardiac function; reduced myocardial fibrosis and hypertrophy; reduced oxidative stress, hypertrophy and fibrosis; decreased cardiomyocyte apoptosis and ferroptosis	[47,391,395,419,420,421,422,423,424,425,426,427,428,429,430,431,432]
Diabetic nephropathy (DN)	NaHS, S-propargylcysteine, GYY4137, Na_2_S_4_	Inhibition of oxidative stress (↑ Nrf2 and antioxidant genes); suppression of RAS activation ((↓ ACE/Ang II/AT_1_R); up-regulation of SIRT1; activation of K_ATP channels; inhibition of NOX4; suppression of NF-κB and MMP-9, and blockade of TGF-β1/Smad3, MAPK/mTOR, Wnt/β-catenin and PI3K/Akt/TLR4 pathway; acts via protein persulfidation	Reduced albuminuria and glomerular injury; attenuation of renal fibrosis; improved renal function	[379,380,382,388,389,433,434,435,436,437,438,439,440,441,442]
Diabetic retinopathy (DR)	NaHS, GYY4137	Inhibition of oxidative stress and mitochondrial dysfunction; reduction in inflammatory cytokines and NLRP3 inflammasome activation; preserves retinal endothelial glycocalyx	Improved retinal neuronal dysfunction, alleviated vascular abnormalities and retinal thickening; protection against hyperglycemia-induced retinal damage	[43,415,417,443]
Diabetes-accelerated atherosclerosis	NaHS, GYY4137, AP39	Improvement of endothelial NO bioavailability via PI3K/Akt/eNOS; reduction in leukocyte adhesion molecules (ICAM-1); inhibition of VSMC proliferation and migration (often via AMPK/mTOR); reduction in NLRP3 inflammasome and NF-κB-driven inflammation; acts via protein persulfidation	Attenuation of atherosclerotic lesion development; improved vascular smooth muscle cell function; reduced plaque formation and foam cell formation	[342,444,445,446,447]
Diabetes-accelerated endothelial dysfunction	NaHS, GYY4137, AP39	Activation of PI3K/Akt/eNOS/NO, reduction in oxidative stress and DNA damage, inhibition of PARP activation; suppression of excessive autophagy via Nrf2/ROS/AMPK signaling; promotion of mitophagy by enhancing PINK1–Parkin interaction and Mfn2 ubiquitination, preserving mitochondrial quality and endothelial viability	Improved endothelium-dependent vasodilation; normalization of vascular reactivity; reduced vascular stiffness	[398,448,449,450,451]
Delayed wound healing in diabetes	3-mercaptopyruvate (3-MP), NaHS, H_2_S-eluting hydrogels, AP39	Increase in pro-angiogenic signaling (including miR-126-3p up-regulation and DNMT1 inhibition in endothelial cells); reduction in oxidative stress and inflammatory responses; improvement of endothelial proliferation and migration	Accelerated wound closure; enhanced angiogenesis; improved collagen deposition; improved neovascularization and tissue repair	[37,40,41,42,400,451,452]
Diabetic neuropathy and neurological complications	NaHS, GYY4137	Inhibition of oxidative stress and inflammation; modulation of ion channels; reduction of iron level and increase in xCT/GPX4/GSH axis; inhibition of proapoptotic pathways	Improved nerve conduction velocity; reduced neuropathic pain; decreased neuropathy and behavioral changes; protection of peripheral nerve structure; decreased apoptosis and ferroptosis	[453,454]

Abbreviations used: Diabetic cardiomyopathy (DCM); NaHS—sodium hydrosulfide; GYY4137—(4-Methoxyphenyl)(morpholino)phosphinodithioic acid; Na_2_S_4_—sodium tetrasulfide; AP39—10-oxo-10-(4-(3-thioxo-3H-1,2-dithiol-5yl)phenoxy)decyl) triphenylphosphonium bromide; Nrf2—Nuclear Factor Erythroid 2-Related Factor 2; NQO1–NAD(P)H:quinone oxidoreductase 1; p38-MAPK—p38 mitogen-activated protein kinase; JNK—c-Jun N-terminal kinase; STAT—Signal transducer and activator of transcription; Wnt—Wingless/Integrated signaling pathway; WISP-1—Wnt1-inducible signaling pathway protein 1; TGF-β1—Transforming growth factor beta 1; NF-κB—Nuclear factor kappa-light-chain-enhancer of activated B cells; TLR4—Toll-like receptor 4; NLRP3—NOD-like receptor family pyrin domain-containing 3; NADPH oxidase (NOX); Mechanistic target of rapamycin (mTOR); GPx4—Glutathione peroxidase 4; GSH—Reduced glutathione; Smad3—SMAD family member 3 (Sons of Mothers Against Decapentaplegic homolog 3); JAK—Janus kinase; PKC—Protein kinase C; ERK1/2—Extracellular signal-regulated kinases 1 and 2; PI3K—Phosphoinositide 3-kinase; Akt (PKB)—Protein kinase B; HO-1—Heme oxygenase-1; RAS—Renin–angiotensin system; ACE—Angiotensin-converting enzyme; Poly (ADP-ribose) polymerase (PARP); Ang II—Angiotensin II; AT_1_R—Angiotensin II type 1 receptor; SIRT1—Sirtuin 1; K_ATP channels—ATP-sensitive potassium channels; MMP-9—Matrix metalloproteinase-9; NO—Nitric oxide; eNOS—Endothelial nitric oxide synthase; ICAM-1—Intercellular adhesion molecule 1; VSMC—Vascular smooth muscle cells; AMPK—AMP-activated protein kinase; PINK1—PTEN-induced kinase 1; Parkin—Parkinson protein 2, E3 ubiquitin–protein ligase; Mfn2—Mitofusin 2; miR-126-3p—MicroRNA-126-3p; DNMT1—DNA methyltransferase 1.

## Data Availability

No new data were created or analyzed in this study. Data sharing is not applicable to this article.

## Supplementary Materials

The following supporting information can be downloaded at https://www.mdpi.com/article/10.3390/antiox15030369/s1. References [19,21,28,29,93,98,104,134,277,469,470,471,472,473,474,475,476,477,478,479,480,481,482,483,484] are cited in the Supplementary Materials.

## Abbreviations

The following abbreviations are used in this manuscript:

3-MST	3-mercaptopyruvate sulfurtransferase
4-HNE	4-hydroxynonenal
5-LOX	5-lipoxygenase
7-DHC	7-dehydrocholesterol
ABCB8	ATP-binding cassette subfamily B member 8
ACSL4	acyl-CoA synthetase long chain family member 4
AGE	advanced glycation end product
AIFM2	apoptosis-inducing factor mitochondria-associated 2
ALOX12	arachidonate 12-lipoxygenase
AMPK	AMP-activated protein kinase
CAT	catalase
CBS	cystathionine β-synthase
cGAS-STING	cyclic GMP-AMP synthase–stimulator of interferon genes pathway
CO	carbon monoxide
COX2	cyclooxygenase-2
CSE	cystathionine γ-lyase
Cys-3S	cysteine-trisulfide
DCM	diabetic cardiomyopathy
DFO	deferoxamine
DHA	dihydroartemisinin
DHODH	dihydroorotate dehydrogenase
DM	diabetes mellitus
DMT1	divalent metal transporter 1
DN	diabetic nephropathy
DR	diabetic retinopathy
Drp1	dynamin-related protein 1
ER	endoplasmic reticulum
ERK	extracellular signal-regulated kinase
ESCRT-III	endosomal sorting complex required for transport-III
ESR1	estrogen receptor alpha
ETC	electron transport chain
Fe^2+^	ferrous iron
Fer-1	ferrostatin-1
FPN	ferroportin
FSP1	ferroptosis suppressor protein 1
FTH1	ferritin heavy chain
FTL	ferritin light chain
FOXO1	forkhead box protein O1
GCH1	GTP cyclohydrolase 1
GCH1-BH4	GTP cyclohydrolase 1–tetrahydrobiopterin
GCL	glutamate-cysteine ligase
GPX	glutathione peroxidase
GPX4	glutathione peroxidase 4
GPDH	glycerol-3-phosphate dehydrogenase
GR	glutathione reductase
GSH	glutathione
GST	glutathione S-transferase
GSS	glutathione synthetase
GYY4137	(4-methoxyphenyl)(morpholino)phosphinodithioic acid
HbA1c	glycated hemoglobin
HG	high glucose
H_2_O_2_	hydrogen peroxide
H_2_S	hydrogen sulfide
HIF-1α	hypoxia-inducible factor 1α
HMGB1	high mobility group box 1 protein
HNO	nitroxyl
HS^−^	hydrosulfide anion
HSNO	thionitrous acid
I/R	ischemia/reperfusion injury
IR–PI3K–Akt	insulin receptor–phosphoinositide 3-kinase–protein kinase B pathway
JAK/STAT	Janus kinase/signal transducer and activator of transcription pathway
JNK/SAPK	c-Jun N-terminal kinase/stress-activated protein kinase
KATP	ATP-sensitive K^+^ channel
Keap1	Kelch-like ECH-associated protein 1
L∙	lipid radical
Lip-1	liproxstatin-1
LOO∙	lipid peroxy radical
LOOH	lipid hydroperoxide
LOX	lipoxygenases
MAPK	mitogen-activated protein kinase
MDA	malondialdehyde
Na_2_S	sodium sulfide
NAC	N-acetylcysteine
NaHS	sodium hydrosulfide
NCOA4	nuclear receptor coactivator 4
NF-κB	nuclear factor kappa B
NINJ1	ninjurin-1
NO	nitric oxide
NOS	nitric oxide synthase
NOD	non-obese diabetic
NOX	xanthine oxidase
NQO1	NAD(P)H quinone dehydrogenase 1
Nrf2	nuclear factor erythroid 2–related factor 2
NTBI	non-transferrin-bound iron
OTUB1	Otubain-1
OXPHOS	oxidative phosphorylation
P–SH	cysteine thiols
P–SO_3_H	sulfonic acid
P–SSH	persulfidated cysteines
PKC	protein kinase C
PPAR	peroxisome proliferator–activated receptor
PRDX2	peroxiredoxin-2
PUFAs	polyunsaturated fatty acids
RAGEs	receptor for advanced glycation end products
RLS	reactive lipid species
RNS	reactive nitrogen species
ROS	reactive oxygen species
RSS	reactive sulfur metabolites derived from H_2_S
RTAs	radical-trapping antioxidants
RSL3	RAS-selective lethal 3
SAS	sulfasalazine
SGLT2	sodium-glucose cotransporter 2
Sirt1	sirtuin 1
SLC7A11	solute carrier family 7 member 11
SOD	superoxide dismutase
STEAP3	metalloreductase
STZ	streptozotocin
Syvn1	E3 ligase synoviolin
T1D	type 1 diabetes
T2D	type 2 diabetes
TBI	transferrin-bound iron
TFR1	transferrin receptor 1
TGF-β1	transforming growth factor β1
TLR4	toll-like receptor 4
TR	thioredoxin reductase
TRP	transient receptor potential family (voltage-gated channels)
TRX	thioredoxin
ubiquinol	CoQ10-H_2_
ubiquinone	coenzyme Q10
xCT	cystine/glutamate antiporter system
XO	xanthine oxidase
ZHX2	zinc fingers and homeoboxes 2
OH	hydroxyl radical
OOH	hydroperoxyl radical
