# Supplementary material for: The Role of Ferroptosis in Diabetes Pathogenesis: Therapeutic Implications of Hydrogen Sulfide and Its Reactive Metabolites
Source: Antioxidants (Basel). 2026 Mar 13;15(3):369. doi: 10.3390/antiox15030369 (PMC13023684; doi:10.3390/antiox15030369)
Supplement: Supplementary file 1 [file antioxidants-15-00369-s001.zip › antioxidants-4179212-supplementary.pdf]

Supplementary table S1: Overview of ferroptosis activators and inhibitors including their mechanism of action, clinical or preclinical application and structural formula.

Agent	Effect	Core mechanism	Clinical / preclinical application in ferroptosis studies	Structural formula	References
Ferrostatin-1 (Fer-1)	Inhibitor	Radical-trapping antioxidant (RTA) that terminates lipid peroxyl-radical chain reactions (suppresses lipid peroxidation downstream of iron).	Canonical "rescue" control to verify ferroptotic death in cell culture and ex vivo tissue models.	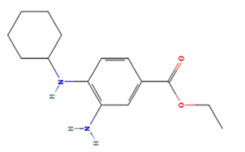	[19,21,28, 277]
Liproxstatin-1 (Lip-1)	Inhibitor	Potent RTA; blocks propagation of phospholipid peroxidation (often used as on-pathway ferroptosis blocker).	Standard rescue control; shown to protect in vivo in settings where ferroptosis is implicated (e.g., glutathione peroxidase 4; GPX4) loss/acute renal injury models).	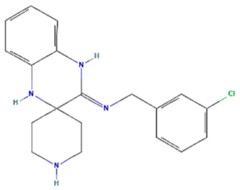	[98,277]
α-Tocopherol / Trolox (vitamin E analog)	Inhibitor	Chain-breaking lipid antioxidant; scavenges lipid radicals in membranes.	Frequently used to suppress/validate lipid peroxidation–driven ferroptotic death (benchmark antioxidant comparators).	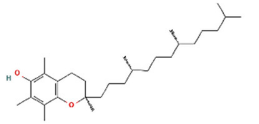 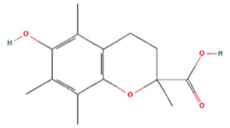	[277,469]
Deferoxamine (DFO)	Inhibitor	Fe chelator that lowers labile iron pool → reduces Fenton chemistry and iron-dependent lipid peroxide formation.	Widely used to block ferroptosis and confirm iron dependence in vitro and in diverse disease models (e.g., neuro/spinal cord injury, tissue injury).	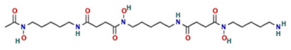	[28,470]

N-acetylcysteine (NAC)	Inhibitor	Restores cysteine/glutathione (GSH) availability → supports GPX4 lipid hydroperoxide detoxification.	Used as a redox/GSH rescue in ferroptosis paradigms (especially upstream of GPX4, e.g., cystine/glutamate antiporter system; xCT inhibition).	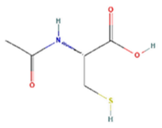	[28,471]
Zileuton (5-lipoxygenase; 5-LOX inhibitor)	Inhibitor	Inhibits 5-LOX–linked lipid oxidation; reduces enzymatic contribution to lipid peroxide buildup (model-dependent).	Used as a pharmacologic probe to test LOX contribution to ferroptosis (notably in neuronal glutamate oxidative toxicity models).	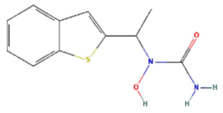	[472,473]
CoQ10 / ubiquinol (supplementation/rescue)	Inhibitor	Expands lipophilic quinone/quinol antioxidant pool; intersects with ferroptosis suppressor protein 1 (FSP1) / dihydroorotate dehydrogenase (DHODH) CoQ-based anti-ferroptotic systems.	Used to test CoQ-axis contribution to ferroptosis resistance and to mitigate ferroptosis phenotypes in tissue-injury models.	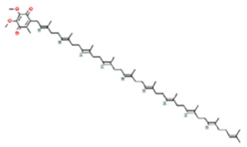	[104,474]
Erastin	Activator	Inhibits system xCT (SLC7A11/SLC3A2) → cystine import ↓ → GSH ↓ → GPX4 capacity ↓ → lipid peroxides accumulate.	Canonical ferroptosis inducer in cell lines and tumor-model studies; used for pathway mapping and sensitization screens.	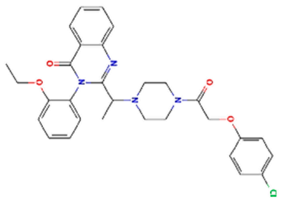	[28,475]
Sulfasalazine (SAS)	Activator	Pharmacologic xCT inhibition → cystine starvation/GSH depletion → ferroptosis sensitization/induction.	Clinically used anti-inflammatory drug; repurposed as an xCT-targeting ferroptosis trigger/sensitizer in cancer and mechanistic studies.	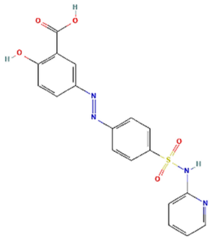	[476]

RSL3	Activator	Used as a "GPX4-pathway" ferroptosis inducer (rapid lipid peroxide accumulation); note: direct GPX4 inhibition has been questioned in some biochemical assays.	Canonical ferroptosis inducer for benchmarking ferroptotic sensitivity and drug-synergy studies.	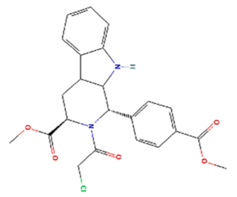	[19,93,478]
ML210	Activator	Covalent/"masked electrophile" GPX4 targeting in cells → lipid peroxide accumulation and ferroptosis.	Widely used GPX4-axis inducer in ferroptosis biology and therapeutic-resistance models.	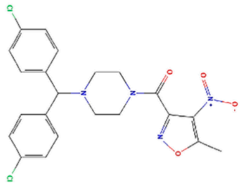	[479]
FIN56	Activator	Promotes GPX4 loss/degradation and perturbs CoQ metabolism → removes key anti-ferroptotic defenses.	Tool inducer used to probe GPX4 stability and mevalonate/CoQ links to ferroptosis.	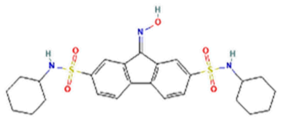	[480]
FINO2	Activator	Endoperoxide-containing oxidant; drives ferroptosis via iron oxidation and functional loss of GPX4 activity with lipid peroxide accumulation.	Tool inducer to study non-canonical initiation mechanisms and structure–activity constraints for ferroptosis triggers.	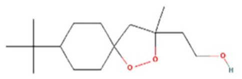	[481]
Sorafenib	Activator	At appropriate concentrations can inhibit xCT and trigger ferroptosis-like death (higher doses may cause mixed death modes).	Clinically approved multi-kinase drug; used in ferroptosis studies (esp. HCC lines) to interrogate xCT-linked ferroptosis and combinatorial sensitization.	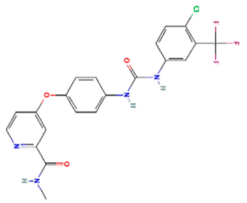	[29,481]

iFSP1 / FSEN1 / icFSP1 (FSP1 inhibitors)	Activator	Inhibit FSP1 CoQ-reducing anti-ferroptotic system → lowers ubiquinol defense → sensitizes to GPX4/xCT stress.	Used to reveal "parallel" ferroptosis defense and to potentiate ferroptosis in cancer models (often combined with GPX4 inhibitors).	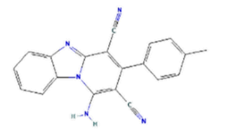 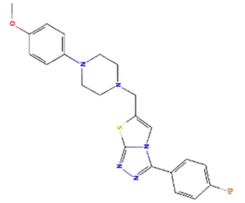 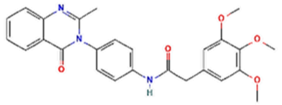	[104,108, 482]
Brequinar / leflunomide (DHODH inhibitors)	Activator	Inhibit DHODH-linked mitochondrial CoQ reduction → weakens mitochondrial anti-ferroptotic defense, especially in GPX4-low settings.	Used to test/target mitochondrial ferroptosis defense axis and induce/sensitize ferroptosis in GPX4-low cancer models.	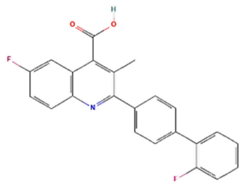 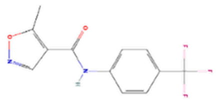	[134]
Dihydroartemisinin (DHA)	Activator	Iron-dependent radical chemistry and redox disruption; multiple studies report ferroptosis contribution (often via xCT/GSH axis and lipid peroxidation).	Used as a ferroptosis-linked trigger or combination partner in cancer models (mechanism and dependence can vary by cell type).	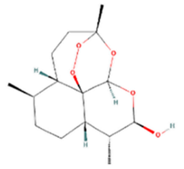	[483,484]

Abbreviations used: Ferrostatin-1 (Fer-1), radical-trapping antioxidant (RTA); liproxtatin-1 (Lip-1); glutathione peroxidase 4 (GPX4); Deferoxamine (DFO); N-acetylcysteine (NAC); lipoxygenase (LOX); glutathione (GSH); cystine/glutamate antiporter (xCT); ferroptosis suppressor protein 1 (FSP1); dihydroorotate dehydrogenase (DHODH); Coenzyme Q (CoQ); solute carrier family 7 member 11 (SLC7A11); solute carrier family 3 member 2 (SLC3A2); sulfasalazine (SAS); RAS-selective lethal small molecule 3 (RSL-3); hepatocellular carcinoma (HCC); dihydroartemisinin (DHA).
